# A global meta-analysis of livestock grazing impacts on soil properties

**DOI:** 10.1371/journal.pone.0236638

**Published:** 2020-08-07

**Authors:** Liming Lai, Sandeep Kumar

**Affiliations:** 1 Department of Agronomy, Hetao College, Bayannur, Inner Mongolia, China; 2 Department of Agronomy, Horticulture and Plant Sciences, South Dakota State University, Brookings, South Dakota, United States of America; Oak Ridge National Laboratory, UNITED STATES

## Abstract

Grazing effects on soil properties under different soil and environmental conditions across the globe are often controversial. Therefore, it is essential to evaluate the overall magnitude and direction of the grazing effects on soils. This global meta-analysis was conducted using the mixed model method to address the overall effects of grazing intensities (heavy, moderate, and light) on 15 soil properties based on 287 papers published globally from 2007 to 2019. Our findings showed that heavy grazing significantly increased the soil BD (11.3% relative un-grazing) and PR (52.5%) and reduced SOC (-10.8%), WC (-10.8%), NO_3_^-^ (-23.5%), and MBC (-27.9%) at 0–10 cm depth, and reduced SOC (-22.5%) and TN (-19.9%) at 10–30 cm depth. Moderate grazing significantly increased the BD (7.5%), PR (46.0%), and P (18.9%) (0–10 cm), and increased pH (4.1%) and decreased SOC (-16.4%), TN (-10.6%), and P (-23.9%) (10–30 cm). Light grazing significantly increased the SOC (10.8%) and NH_4_^+^ (28.7%) (0–10 cm). Heavy grazing showed much higher mean probability (0.70) leading to overgrazing than the moderate (0.14) and light (0.10) grazing. These findings indicate that, globally, compared to un-grazing, heavy grazing significantly increased soil compaction and reduced SOC, NO_3_^-^, and soil moisture. Moderate grazing significantly increased soil compaction and alkalinity and reduced SOC and TN. Light grazing significantly increased SOC and NH_4_^+^. Cattle grazing impacts on soil compaction, SOC, TN, and available K were higher than sheep grazing, but lower for PR. Climate significantly impacted grazing effects on SOM, TN, available P, NH_4_^+^, EC, CEC, and PR. Heavy grazing can be more detrimental to soil quality based on BD, SOC, TN, C: N, WC, and K than moderate and light grazing. However, global grazing intensities did not significantly impact most of the 15 soil properties, and the grazing effects on them had insignificant changes over the years.

## Introduction

Livestock production, the largest land-use sector on Earth, causes severe problems such as water pollution, global warming, and soil degradation in some regions [1, 2]. Soil degradation due to livestock grazing is an emerging global problem [3]. Livestock grazing degraded an estimated 20–35% of the world’s permanent pastures [4]. The four factors of livestock grazing impacting soil quality [5] include the type of livestock [6, 7], grazing intensity [7], level of plant productivity [8], and evolutionary history of grazing [9]. Grazing intensity affects soil structure, function, and capacity impacting soil organic carbon (SOC) storage in livestock-plant-soil systems [10]. Grazing intensity also impacts the range of ecosystem services such as nutrient retention, water storage, pollutant attenuation [11]. Although many studies reported on the impact of grazing intensity on soil properties [12–14], the individual experimental design and different local conditions resulted in inconsistent findings on select soil properties [15]. These inconsistent findings led to increased debates about the effectiveness of the grazing system [16]. The studies, for instance, reported a range of findings, including increased, decreased, or no change to the soil carbon (C) and nitrogen (N) levels [12]. These different results may mask some problems and mislead the public, preventing authorities or governments from addressing true soil problems. This confusion may lead to serious consequences such as increases in soil degradation and reduction in livestock production in the long-term. Therefore, this study analyzed all related individual and scattered studies worldwide using synthesized analysis method to find the overall magnitude and direction of grazing effects on different soil properties.

Meta-analysis is a mature synthesized analysis method and a powerful quantitative approach to address inconsistent ecological studies with differing directions and outcomes [15]. Meta-analysis explores relationships that cannot be investigated in primary studies, such as links between treatments and environmental factors [17]. Other studies used meta-analysis to address major ecological issues did not assess the overall global grazing effects on soil properties such as a wide range of possible response variables, grazing types, and environmental conditions in recent decades [12, 15, 18, 19]. Furthermore, although the total annual meat and milk from cattle, sheep, and goats in the world increased from 1961 to 2018, the total annual meat and milk production increased at a faster rate (greater than 23 million tons and 180 million tons, respectively) since 2003. Additionally, the global annual area of permanent meadows and pastures decreased by less than 3.4 million ha since 2003 [20]. Increased meat and milk production and decreased permanent grasslands indicate increased grazing pressure on the livestock grazing lands since 2003. The increased grazing pressure likely led to increases in soil property changes. The studies published since 2007 could cover the data of the grazing effects on the soil properties since 2003.

Therefore, utilizing comprehensive meta-analytical approach based on publications from 2007 to 2019, the objective of this study was to address the overall effects of global grazing intensities on 15 soil properties: soil bulk density (BD), penetration resistance (PR), SOC, total nitrogen (TN), C: N ratio, ammonium (NH_4_^+^), nitrate (NO_3_^-^), microbial biomass carbon (MBC), microbial biomass nitrogen (MBN), available phosphorus (P) and potassium (K), cation exchange capacity (CEC), pH, electrical conductivity (EC), and water content (WC). The findings from this study shed new light on the universal relationships between grazing intensity and soil properties and inform sustainable grazing practices from global, temporal, and environmental perspectives.

## Materials and methods

### Data compilation and definition of effect size for meta-analysis

**Fig 1:**
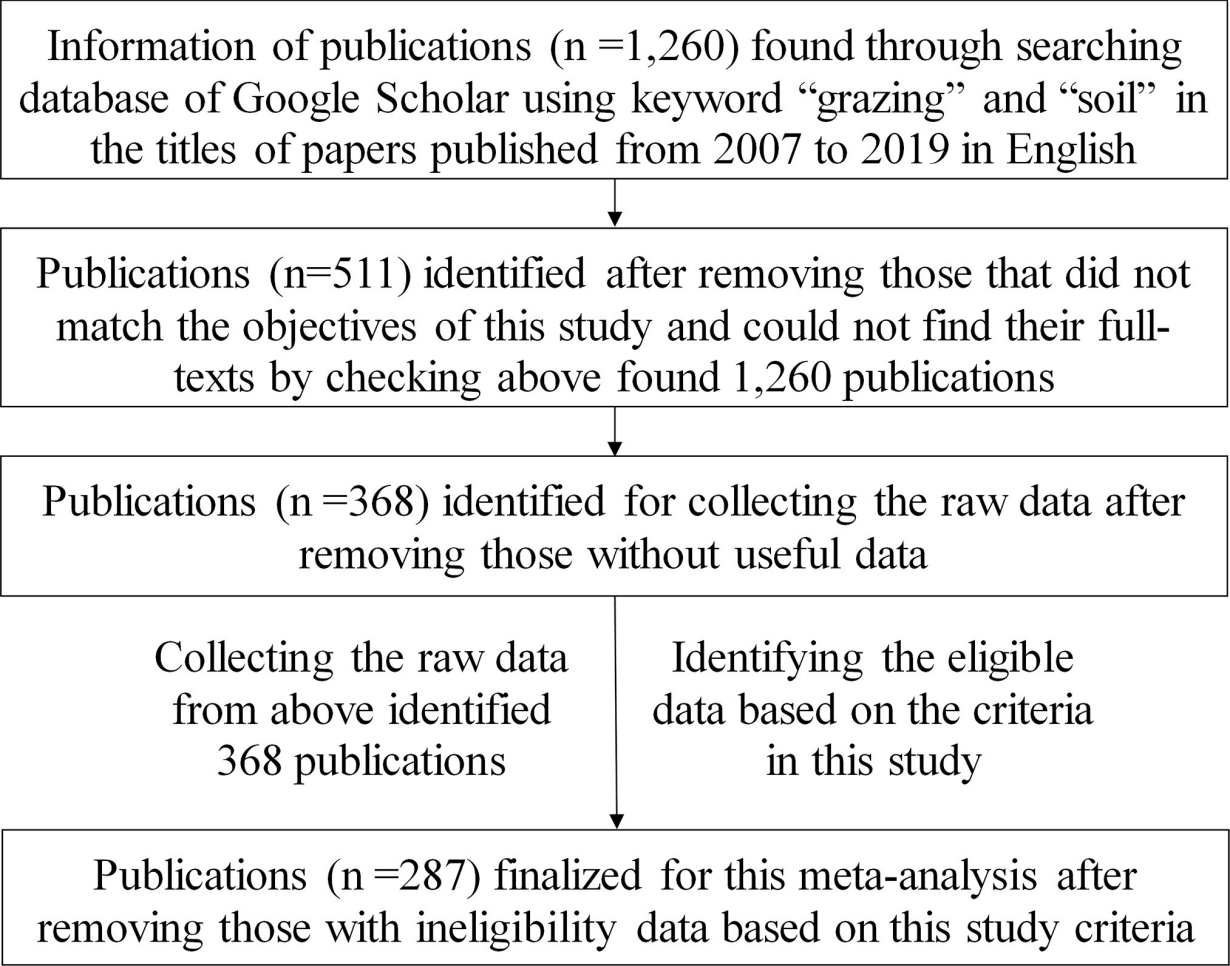
A flow diagram showing the process and methods for publication search and data collection in this meta-analysis.

287 publications were selected from 1,260 referred English papers published from 2007 to 2019. The publications were discovered by searching Google Scholar using the keywords “grazing” and “soil” in the titles of papers to collect data (S1 Dataset) and compile dataset (S2 Dataset). All raw data were extracted from tables and digitized graphs of the original publications using WebPlotDigitizer software (Version 3.8 for Desktop). A flow chart of process and methods for publication search and data collection are presented in Fig 1.

The raw publication data collected for this meta-analysis met the following criteria: (i) Soil property data under various grazing intensities (≥3) and the un-grazing from different field sites at 0–30 cm depth were collected. Because grazing data cannot be compared to un-grazing at the same time, this study excluded temporal comparisons data (before and after grazing) of the same site. (ii) Data at sites grazed by domestic animals such as cattle (*Bos Taurus* L.), sheep (*Ovis aries* L.) (including goat and deer), or their mixture were selected. (iii) This study defined non-adjacent sites to have a distance greater than 46 km (an approximate distance of 25’ in latitude range). Non-adjacent site data were considered independent and included in the database. Adjacent site data were only limited to one site to avoid dependent data with different sites. (iv) Data collected within a year for each site were averaged [12]. Data collected over multiple years could be time-dependent (e.g., autocorrelation). As a result, only the data from the most recent year were included. (v) Site information including grazing intensity, overgrazing, grazing animals, grazing period, land-use, sampling year, soil depth, latitude, longitude, altitude, country, soil texture, mean annual precipitation, and mean daily temperature were all collected. Missing information such as latitude, longitude, altitude, temperature, and precipitation in some publications were retrieved from the websites (https://www.ncdc.noaa.gov/cdo-web/datasets; http://www.worldclimate.com/; https://www.freemaptools.com/elevation-finder.htm; https://www.google.com/maps/) based on the information of study locations. The grazing intensity level (heavy, moderate, or light) and overgrazing (or non-overgrazing) were defined based on the authors’ original studies. Due to the wide range of livestock types and units used in different studies, the intensity has various stocking rates. This study also defined three types of the lands: grassland (GLD), grassland with trees (GLT) (including forests for grazing), and integrated crop-livestock system (ICLS).

This meta-analysis re-coded and re-arranged the raw data based on the following principal rules: (i) This study categorized four levels of grazing intensities based on the original publications: heavy, moderate, light, and zero (un-grazing). For studies with more than four levels, the grazing intensities were recategorized into the four levels based on their results. For studies without un-grazing data, the lowest level (generally light or very light grazing) was treated as the un-grazing level. (ii) The selected studies defined the 0–10 cm depth as the topsoil layer and the 10–30 cm depth as other increments. Data for more than 30 cm depth were excluded due to the small sample size. Data reported in different sub-depths within the above-defined increments were interpolated to the corresponding depth by summing the soil property data in sub-depths together [12]. Therefore, this study established sampling increments of 0–10 cm and 10–30 cm depths for BD, SOC, TN, C: N, pH, and P. Due to the lack of data for 10–30 cm increment, this study only examined the remaining soil properties for 0–10 cm depth (WC, NH_4_^+^, NO_3_^-^, K, PR, EC, CEC, MBC, and MBN) (S1 Table). (iii) Sandy clay, sandy clay loam, sandy loam, loamy sandy, and sand were re-coded as “sandy” due to high sand contents. Other soil types with lower sand contents were re-coded as “other” for analyzing the grazing effects on soils.

Partial values were calculated by using the following equations. (i) For some cases with SOM but no SOC data, the SOM values were converted into SOC using equation [21]: SOC = SOM × 0.58. (ii) In studies with SOC stock (Mg ha^−1^) but no SOC (g kg^-1^) data, the SOM stock were converted into SOC using equation [22]: SOC = SOC stock/(BD × d); where d = soil depth (m); BD = soil bulk density (Mg m^-3^). A similar equation was used for converting TN storage to TN concentration [23]: TN = TN storage/(BD × d). For some MBC and MBN stocks, the same equation was used for converting these storages to concentrations. (iii) The C: N ratio was calculated using the equation: C:N = SOC/TN (the units of SOC and TN must be the same). The equation was also used for calculating SOC or TN if the C: N and TN or SOC were given in the studies.

Effect size (ES) in this meta-analysis was defined as the natural log of the response ratio as a metric for the response of the soil property to grazing. For a given soil property X, the effect size was calculated based on the equation [12, 15, 18]:

$$ES_{x}=\mathrm{Ln}\left(\frac{X_{g}}{X_{ug}}\right)=LnX_{g}-Ln(X_{ug})$$

where *ES*_*x*_ is the effect size of X; *X*_*g*_ is the value of X in the grazing group; *X*_*ug*_ is the value of X in the un-grazing group. This study examined a total of 15 soil properties.

**Fig 2:**
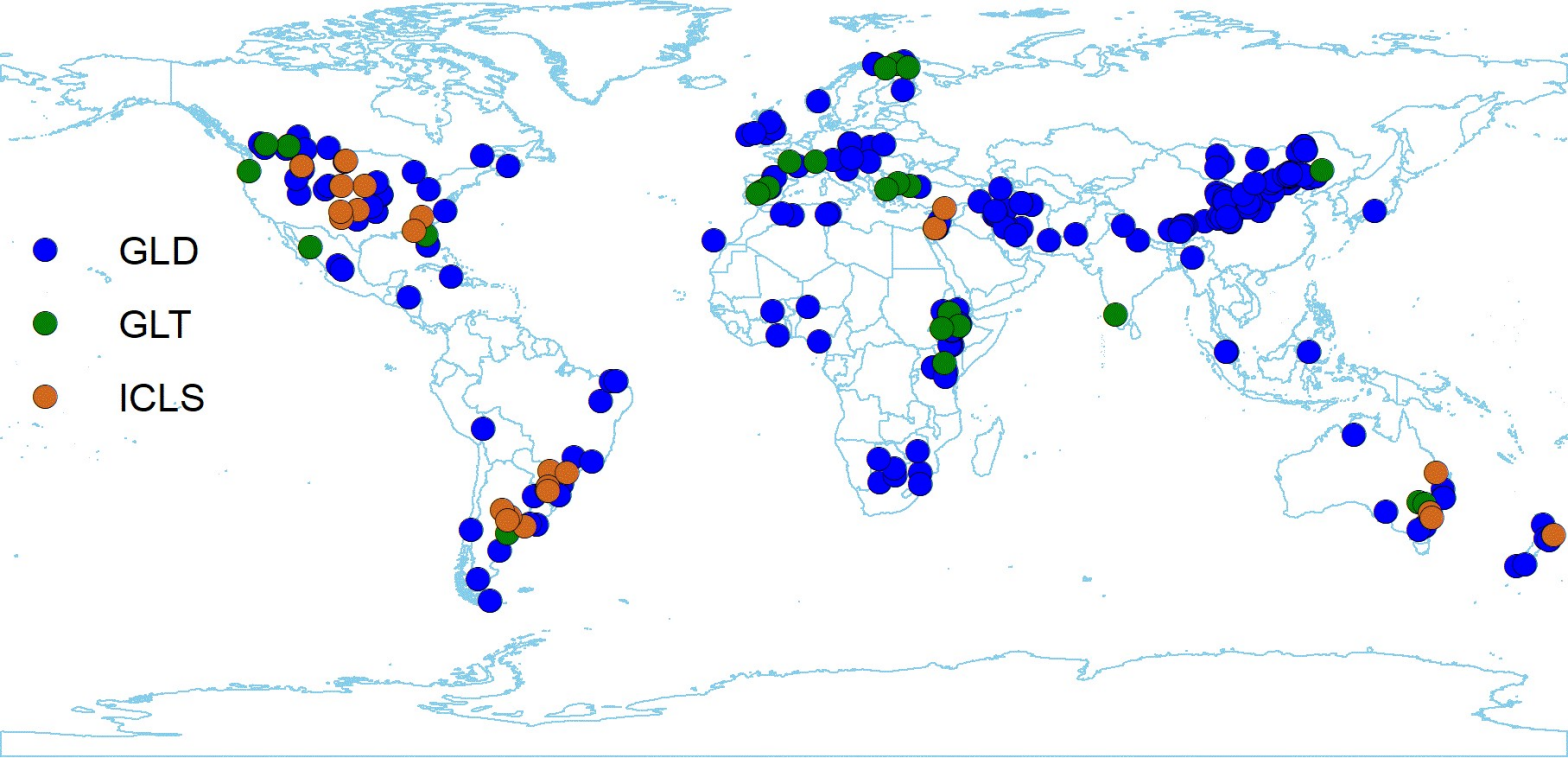
Total of 276 locations of study sites in this meta-analysis based on 287 publications from 2007 to 2019. GLD, grassland; GLT, grassland with trees; ICLS, integrated crop-livestock system.

The above 33 variables (15 variables of soil property effect sizes, 14 variables of site information, and 4 variables of publication information) were compiled as a database in an Excel file (S2 Dataset). S1 Table summarized the basic information of the database and displayed the years of sampling investigation from 1998 to 2018. The data stemmed from 278 distributed locations across 52 countries from 287 independent publications (Fig 2).

### Meta-analysis method

This study used the mixed model method in PROC MIXED in SAS9.4 to conduct this meta-analysis [24]. The mixed model method is suitable for analyzing differences between groups of experiments when the groups are not expected to be internally homogeneous [25]. One potential disadvantage of using randomization tests (resampling method) is the lack of separation between the two sources of variance: within-study sampling error and between-study variation in true effects. The mixed model was designed to address this limitation [25]. The 21 mixed models (15 for the 0–10 cm depth and 6 for the 10–30 cm depth) were built using the 21 soil property effect sizes as dependent variables. Grazing intensity (main effect), soil texture, land-use, grazing animal, latitude, altitude, precipitation, temperature, grazing period, and sampling year were 10 independent variables used as the fixed effects, and the study ID was used as the random effect in the 21 mixed models. The latter 9 variables as the fixed effects were regarded as covariates for adjusting the mean effect sizes among the 3 grazing intensities [26]. For each soil property and each grazing intensity, if the number of effect sizes is less than 10, the mixed models excluded those effect sizes and used the data under the other two grazing intensities instead. In this study, 6 soil properties under light grazing did not have enough data points and were excluded from the models. For the same reason, land-use variable and grazing animal variable for some soil properties also did not have enough data points and were excluded from the models. This meta-analysis required data transformation to achieve optimal mixed models. The transformation methods were determined using the Box-Cox method [27, 28] using SAS9.4 [24]. Variance inflation factors (VIFs) were used to detect the presence of multicollinearity among the independent variables [29] using SAS9.4 [24]. For VIF greater than 10, the model faces the multicollinearity problem. As a result, the models in this study were restricted to VIF values of less than 10. The mixed models used the SAS algorithm to estimate the least-square means (LS-means) of soil property effect sizes and confidence intervals (CIs) of the 3 grazing intensities [30]. The LS means were adjusted by covariates and random effects in the mixed models. The LS-means and CIs of the soil property effect sizes indicate the grazing intensity effects on soil properties. The LS-means and CIs were reported as the percentage change estimated by (*e*^*ES*^ − 1) × 100% [12]. For other independent character variables, we cannot use their LS-means to explain the effects on soil properties because the dependent variables were only grazing effect size. For the 9 covariates in the mixed models, the positive (+) or negative (-) signs of coefficients indicate positive or negative impacts of these variables on soil property effect sizes. These positive and negative impacts represent the effects of interaction between these variables and the grazing intensity on the soil properties. For the coefficients of independent variables, the impacts are significant if the p-values of F-testing is less than 0.10 (* represents the significant impacts in this study). However, the coefficients of independent variables cannot be interpreted as the percentage increase or decrease in effect sizes of soil properties. This is due to the dependent variables (i.e. effect sizes) required different transformation algorithms when using the Box-Cox method to build the 21 models.

Furthermore, we built the binomial logistic models using SAS 9.4 to predict the probabilities of the 3 grazing intensities resulting in overgrazing [24]. We determined significance at α = 0.10 for all models due to different limited degrees of freedom for different soil property effect sizes (S1 Table) [31, 32]. See S1 File under Supporting Information for more details.

## Results

### Global grazing effects on soil properties

**Fig 3:**
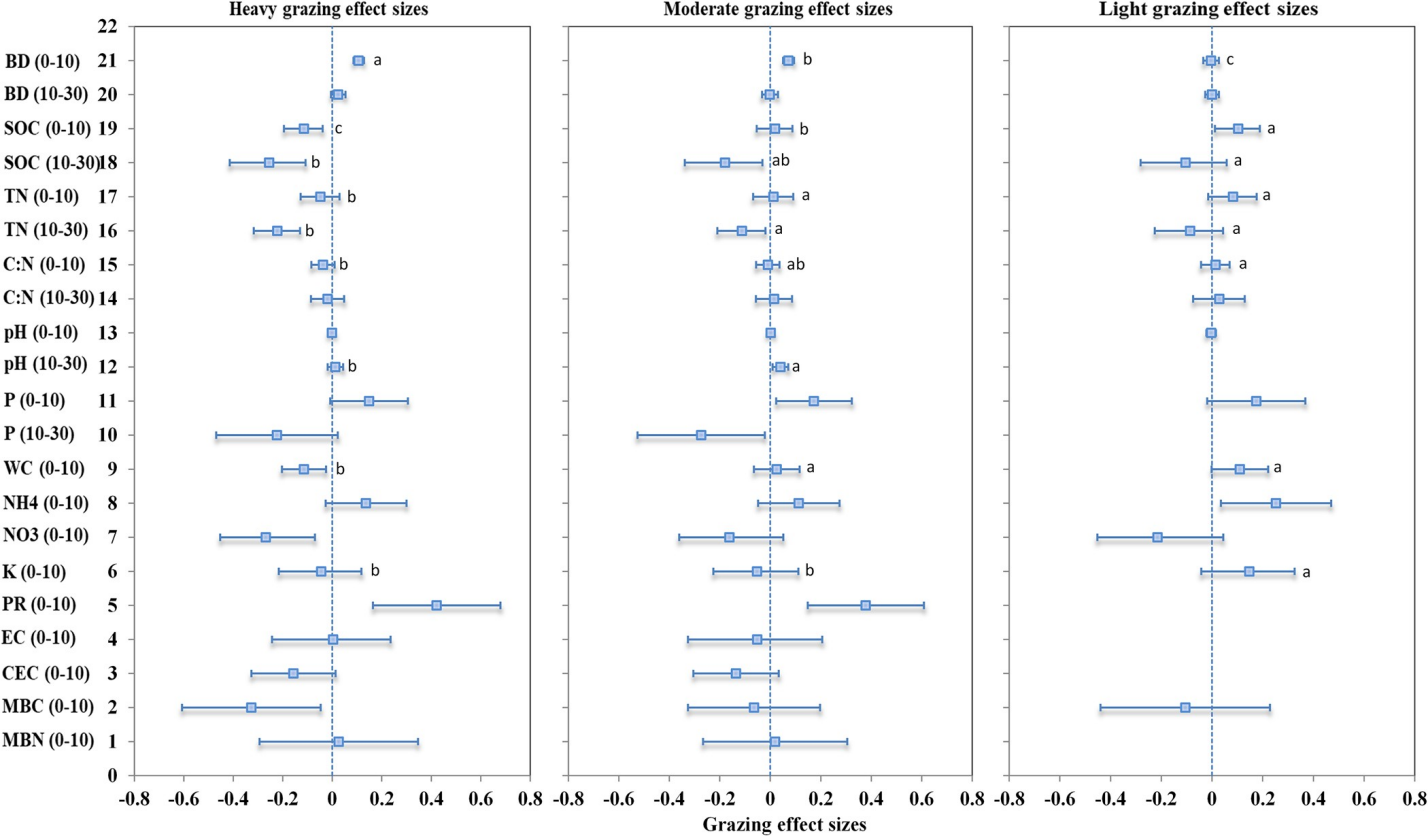
Global grazing effect sizes of 15 soil properties under 3 grazing intensities at 2 soil depths. Bars represent the means and their ranges of 90% confidence intervals (CIs). The vertical dashed line was drawn at the grazing effect size = 0. Means within the same row followed by different small letters are significantly different at P<0.10 for the grazing intensities.

The global grazing effects on 15 soil properties for the 0–10 and 10–30 cm depths are presented in Fig 3. The LS-means and CIs percentage change of grazing effect sizes for the 15 soil properties are presented in S2 Table. Compared to un-grazing, the heavy grazing intensity significantly increased the soil BD [an average LS-means of 11.3% (90% CI: 8.9–13.7%, the same format below)] and PR [52.5% (17.9–97.4%)] at the 0–10 cm depth. Heavy grazing intensity also significantly decreased the SOC [-10.8% (-17.7- -3.8%)] at the 0–10 cm depth and SOC [-22.5% (-33.9- -10.2%)] at the 10–30 cm depth, TN [-19.9% (-27.2- -12.2%)] at the 10–30 cm depth, and WC [-10.8% (-18.4- -2.5%)], NO_3_^-^ [-23.5% (-36.4- -6.7%)], and MBC [-27.9% (-45.5- -4.6%)] at the 0–10 cm depth.

Moderate grazing significantly increased the soil BD [7.5% (5.2–9.9%)], PR [46.0% (16.0–83.7%)], and P [18.9% (2.3–38.1%)] at the 0–10 cm depth and pH [4.1% (0.9–7.4%)] at the 10–30 cm depth. Moderate grazing significantly decreased the SOC [-16.4% (-28.7- -3.0%)], TN [-10.6% (-18.9- -1.9%)], and P [-23.9% (-40.9- -2.1%)] at the 10–30 cm depth.

Light grazing significantly increased the SOC [10.8% (1.1–20.8%)] and NH_4_^+^ [28.7% (3.6–60.0%)] at the 0–10 cm depth.

The mean BD effect size under heavy grazing (i.e., a mean change in BD by heavy grazing compared with un-grazing: 11.3%, the same format below) was significantly higher than moderate (7.5%) and light grazing (-0.4%) at the 0–10 cm depth. However, the mean SOC (-10.8 and -22.5%) and TN (-4.7 and -19.9%) effect sizes under heavy grazing were significantly lower than moderate grazing [SOC (1.9 and -16.4%) and TN (1.3 and -10.6%)] and light grazing [SOC (10.9 and -9.9%) and TN (8.6 and -8.3%)] at the 0–10 and 10–30 cm depths, respectively. The mean C:N (-3.6%), WC (-10.8%), and K (-4.3%) effect sizes under heavy grazing were significantly lower than moderate grazing [C: N (-0.9%), WC (2.6%), and K (-5.1%)] and light grazing [C: N (1.4%), WC (11.6%), and K (15.8%)] at the 0–10 depth. The mean pH effect size under heavy grazing (1.3%) was significantly lower than moderate grazing (4.1%) at the 10–30 depth (Fig 3 and S2 Table).

### Impacts of environmental factor and time on the global grazing effect sizes of soil properties

The coefficients of soil texture, land use, animal, grazing period, latitude, altitude, precipitation, temperature, and year in the mixed models are presented in Table 1 and S3 Table.

**Table 1:** The coefficients of the soil texture (Sandy vs others), animal (Cattle vs. sheep), grazing period (Period), precipitation (Prcp), temperature (Temp), and sampling year (Year) in the 21 mixed models for the 0–10 and 10–30 cm depths. The complete covariate coefficients are presented in S2 Table.

Effect sizes	Sandy vs Others^a^	Cattle vs Sheep	Period (1/100)	Prcp (1/10000)	Temp (1/100)	Year (1/100)
	0–10 cm depth					
BD	-0.019*	-0.004	0.043	0.160	-0.069	0.044
SOC	-14.45	65.83*	-125.4*	52.10	-4.694	-18.20
TN	-0.015	5.567*	-6.699	76.98*	-13.57	44.14
C:N	-0.183	0.237	-0.385	0.390	-1.105	-3.544
pH	-0.002	-0.013	0.030	0.033	-0.020	0.054
P	0.037	0.111	-0.439	3.990*	-2.614*	-0.759
WC	0.021	0.069	-0.472*	0.150	0.296	-0.553
NH_4_^+^	-0.138	0.236	0.184	2.360	2.963*	-0.553
NO_3_^-^	-0.141*	-0.106	0.158	1.190	0.312	0.554
K	2.156*	2.266*	-0.435	22.94	-2.698	6.288
PR	0.031	-0.781*	0.982	6.520*	-4.222*	-4.955
EC	-3.177	1.855	-10.60	4.140	31.00*	37.53
CEC	0.065	-	-0.450	2.170	2.126*	-1.134
MBC	0.046	0.026	0.272	-1.900	1.531	1.251
MBN	0.346	-0.202	1.705	1.840	0.534	0.047
	10–30 cm depth					
BD	-0.011	0.031*	-0.080*	-0.200	0.050	0.069
SOC	-0.114	1.454	-6.916	116.5*	-9.453	2.203
TN	1.884	1.759	17.56	75.97	40.74	1.892
C:N	0.087	-0.241	0.163	2.120	-3.030	-3.429
pH	-0.002	-0.047	0.035	0.034	-0.114	-0.297
P	0.753*	0.106	2.428*	-0.100	-0.881	-5.504

^a^Others include silty and clayey soils.

*indicates that the independent variables as covariates significantly impacted the effect size of soil property in the mixed model (P<0.10). However, the coefficients of the independent variables cannot be used to interpret how many percentages they can increase or reduce the effect sizes of soil properties because the dependent variables (i.e., effect sizes of soil properties) were transformed using different algorithms based on the Box-Cox method when building these models.

Data from the 0–10 cm depth showed that (i) the coefficients of soil texture (Sandy vs others) in the mixed models for the effect size of BD and NO_3_^-^ as dependent variables were -0.019 and -0.141, respectively (Table 1). The negative (-) coefficients indicated that sandy soils had significantly lower effect sizes of BD and NO_3_^-^ than other soil textural classes. In the same way, the positive (+) coefficient indicated that sandy soil had a significantly higher K effect size (coefficient of sandy vs. others in the mixed model for K effect size was 2.156, the same format below) than other soil textural classes (Table 1). Similarly, (ii) ICLS had significantly higher effect sizes of BD (0.022) than GLD. GLT had significantly higher effect sizes of BD (0.031) and SOC (75.90) than ICLS (S3 Table). (iii) Cattle grazing had significantly higher effect sizes of SOC (65.83), TN (5.567), and K (2.266) than sheep grazing. Cattle grazing had significantly lower PR effect size (-0.781) than sheep grazing (Table 1). Land grazed by mixed cattle and sheep had significantly lower effect sizes of BD (-0.022), NO_3_^-^ (-0.275), and PR (-0.929) than land grazed by sheep alone (S3 Table). (iv) The grazing period had significant negative impacts on the effect sizes of SOC (-125.4/100) and WC (-0.472/100) (Table 1). (v) Precipitation had significant positive impacts on the effect sizes of TN (76.98), P (3.99), and PR (6.52/10000). The temperature had significant positive impacts on the effect sizes of NH_4_^+^ (2.963), EC (31.00), and CEC (2.126) and significant negative impacts on the effect sizes of P (-2.6140, and PR (-4.222/100) (Table 1). (vi) Latitude had significant negative impacts on the P effect size (-4.72) but significant positive impacts on the EC effect size (112.8/1000) (S3 Table). (vii) Altitude had significant negative impacts on the effect sizes of C: N (-1.8) and pH (-0.1/10000) (S3 Table). (vii) The sampling year had no significant impacts on the effect sizes of the soil properties. However, the effects still exist, i.e., the sampling year positively impacted the effect sizes of BD, TN, pH, NO_3_^-^, K, EC, MBC, and MBN, but negatively impacted the effect sizes of SOC, C: N, P, WC, NH_4_^+^, PR, and CEC (Table 1).

Data from the 10–30 cm depth showed that (i) sandy soil had significantly higher P effect size (0.753) than other soil textural classes (Table 1). (ii) ICLS had significantly higher BD effect size (0.022) than GLD (S3 Table). (iii) Cattle grazing resulted in significantly higher BD effect size (0.031) than sheep grazing (Table 1). (iv) The grazing period had negative impacts on the BD effect size (-0.080) but positive impacts on the P effect size (2.428/100) (Table 1). (v) Precipitation had a positive impact on the SOC effect size (116.5/10000) (Table 1). (vi) Even though the sampling year did not significantly impact all 6 soil property effect sizes, it positively impacted the effect sizes of BD, SOC, and TN but negatively impacted the effect sizes of C: N, pH, and P (Table 1).

### Estimated probabilities of the 3 grazing intensities leading to overgrazing

The 0–10 and 10–30 cm depths results from the logistic models showed high probabilities of heavy grazing intensity leading to global overgrazing (Table 2 and S1 and S2 Figs). In the 0–10 cm depth, the mean (90% CIs) of the 15 predicted probabilities of heavy grazing intensity resulting in overgrazing was 0.685 (0.523–0.819). However, the moderate and light grazing resulted in much lower mean probabilities (90% CI) with 0.099 (0.041–0.218) and 0.069 (0.016–0.246), respectively. The 10–30 cm depth data mirrored similar results.

**Table 2:** Means of the 15 predicted probabilities and their 90% CIs at the 0–10 cm depth and the 6 at the 10–30 cm depth for each grazing intensity resulting in overgrazing based on the logistic model (the dependent variable is binary “overgrazing” and “non-overgrazing”).

Grazing intensities	Predicted probabilities		
	Mean	Lower	Upper
Heavy grazing (0–10 cm)	0.685	0.523	0.819
Moderate grazing (0–10 cm)	0.099	0.041	0.218
Light grazing (0–10 cm)	0.069	0.016	0.246
Heavy grazing (10–30 cm)	0.714	0.541	0.842
Moderate grazing (10–30 cm)	0.185	0.084	0.364
Light grazing (10–30 cm)	0.129	0.027	0.443

## Discussion

### Global effects of livestock grazing on soil properties

This meta-analysis showed that compared to un-grazing, the heavy and moderate grazing significantly increased soil BD and PR and reduced SOC and TN. Heavy grazing significantly decreased NO_3_^-^, WC, and MBC. Moderate grazing significantly increased pH and P at the 0–10 cm depth but reduced P at the 10–30 cm depth. Light grazing significantly increased SOC and NH_4_^+^. When compared to un-grazing, heavy grazing resulted in significantly greater increase in soil BD than moderate and light grazing. Additionally, heavy grazing resulted in significantly greater reduction in SOC, TN, C: N, WC, and K than moderate and light grazing (Fig 3 and S2 Table). High BD and PR and low SOC, MBC, TN, NO_3_^-^, NH_4_^+^, K, P, and WC indicate poor soil quality [22, 33, 34]. As a result, heavy grazing suggested greater detrimental impacts on soil quality than moderate and light grazing. These results are mainly contributed to grazing animal activities.

Animal activities affect soils in three main ways: (i) trampling soils, (ii) grazing plant materials and roots, and (iii) adding animal excretes to soils. Animal grazing can eliminate standing dead plant biomass, thus decreasing SOC [12]. Animal trampling can accelerate the physical breakdown of plant materials. This results in faster litter decomposition [12], reducing carbon sequestration and hence SOC [35]. Animal grazing also reduces plant cover on soil surface [36, 37] leading to greater water erosion [38, 39] and reducing SOC in topsoil. The reduction in SOC can increase soil BD [40] and reduce soil organic N [41], consequently reducing soil NH_4_^-^ and NO_3_^-^. Moreover, the reduced plant cover removes a layer of protection on the soil surface and can increase amplitude in soil moisture [42, 43], leading to decreased soil WC [44] and increased soil BD. Animal trampling also reduces soil porosity and water circulation, resulting in increased soil BD [45]. Since PR is positively related to BD and negatively to WC [46], the increase in BD and reduction in WC by grazing can increase PR.

Furthermore, animal trampling and grazing can lower litter quality for soil microbes and fauna [47], leading to decreased microbial growth and activity, MBC, and MBN [48], and TN. Additionally, trampling and grazing can reduce the biomass and decomposition rate of litter and plant roots [49, 50]. This biomass reduction lowers the soil N nutrients (NH_4_^-^ and NO_3_^-^) derived from litter and roots, subsequently reducing soil TN. While the grazing animal excretes can add N to the soils [14], the amount of N deposition is minor [51]. Therefore, grazing could overall reduce soil TN. Additionally, animal excretes could increase the soil pH [52, 53] and add available P to topsoil. However, during the microbial breakdown of plant materials, phosphorus is released slowly [54].

Compared to moderate and heavy grazing, light grazing has increased levels of SOC and NH_4_^+^ (Fig 3), which could be attributed to more plants and less animal excretes on the soil. With sufficient plants and materials under light grazing, the soils could have increased SOC and soil organic N [12, 41], resulting in increased soil NH_4_^+^.

This study also found that heavy grazing did not significantly impact C: N, pH, available P and K, NH_4_^+^, EC, CEC, and MBN. Moderate grazing did not significantly impact C: N, available P and K, NH_4_^+^, NO_3_^-^, EC, WC, CEC, MBC, and MBN. Light grazing did not significantly impact 13 of 15 soil properties (Fig 3). These findings indicated that heavy grazing did not affect the balance of soil C and N, soil acidity and alkalinity, soil salinity, soil cation exchange capacity, available P and K, and microbial biomass. Moderate and light grazing did not influence most of the 15 soil properties.

Compared to moderate and light grazing, heavy grazing generated higher trampling intensity and diminished more plant cover at surface soil, resulting in increased BD and reduced SOC, TN, C: N, WC, and K (Fig 3). We found that heavy grazing has a high potential to result in overgrazing (mean predicated probability = 0.685 for the 0–10 cm depth and 0.714 for the 10–30 cm depth). Moderate and light grazing showed a low potential to result in overgrazing (mean predicated probability ≤ 0.185) (Table 2). Therefore, heavy grazing could have much more detrimental impacts on soil quality than moderate and light grazing.

Compared to previous meta-analyses that were limited by region or number of analyzed soil properties [11, 12, 19, 55–58], this study provides more insight into global grazing impacts on the soil by combining large-scale data of 15 soil properties.

### Global impacts of interactions between the grazing and main environmental factors on soil properties

This meta-analysis found that 5 of 9 independent variables as covariates significantly impacted 4 or 5 out of 21 effect sizes of soil properties at the 0–10 and 10–30 cm depths. The other 4 independent variables significantly impacted 0–3 out of 21 effect sizes (Table 1 and S3 Table). As a result, we considered soil texture, animal (cattle vs. sheep), grazing period, precipitation, and temperature as the 5 main environmental factors that impacted global grazing effects on soil properties.

**Fig 4:**
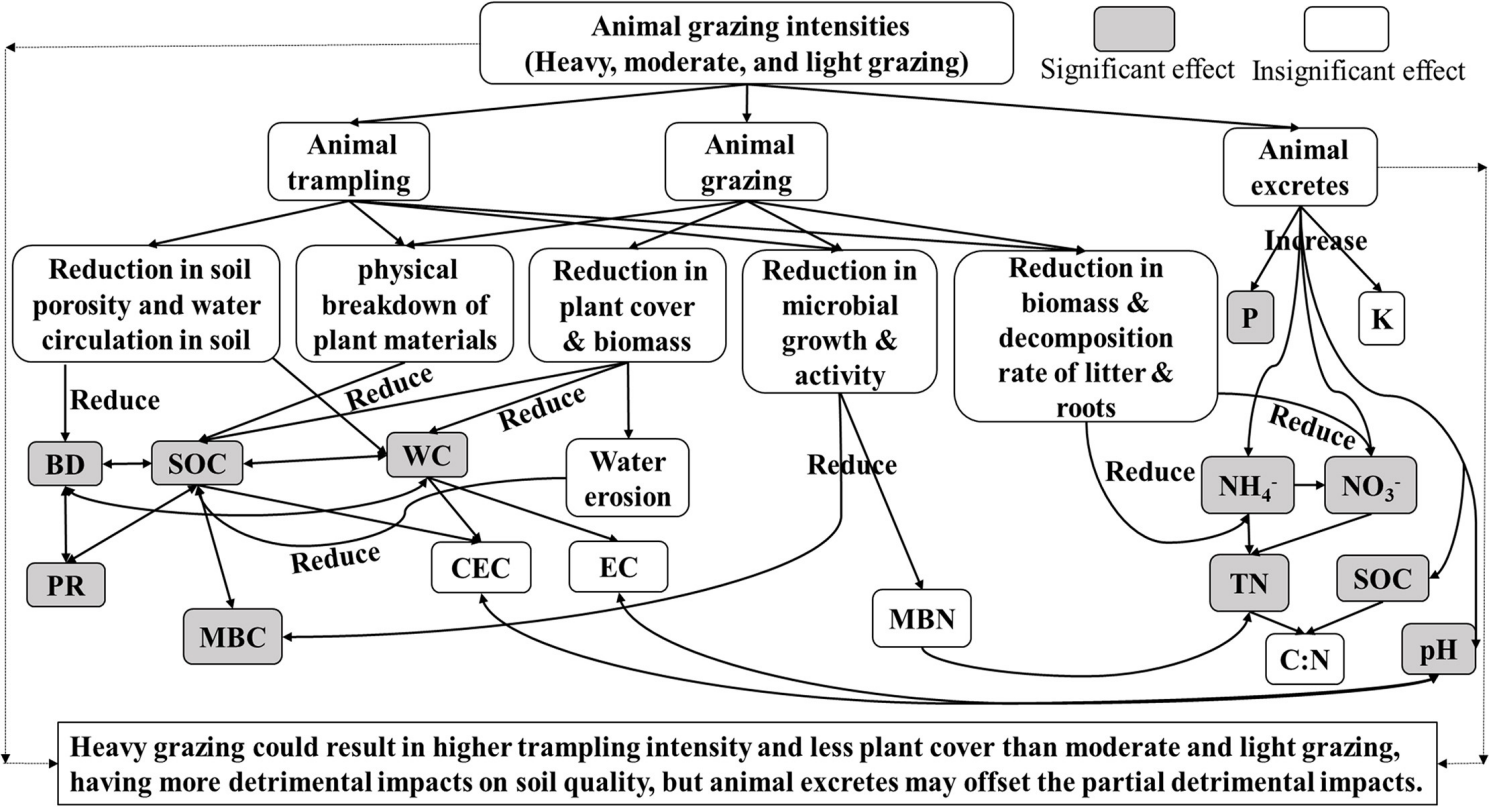
Proposed livestock grazing impacts on the 15 soil properties.

Soil texture (sandy vs. other) significantly impacted the effect sizes of BD, NO_3_^-^, K, and P (Table 1). This finding indicated that the impacts of grazing on BD and NO_3_^-^ (compared to the un-grazing) in sandy soil were significantly lower than other soils (silty and clayey soils). However, the impacts on P and K in sandy soil were higher than other soils. The lower impacts of grazing on BD in sandy soil, compared to the other soils, is primarily because the sandy soil is more difficult to be disturbed by grazing animals than the other soils, resulting in less change in soil porosity and water circulation by animal trampling [45] (Fig 4). The lower impacts of grazing on NO_3_^-^ at the sandy soil, compared with the other soils, is primarily because the animal trampling less likely reduces plant biomass and decomposition rate of litter and roots at the sandy soil (Fig 4). Moreover, the higher impacts of grazing on available P (10–30 cm) and K (0–10 cm) at the sandy soil than the other soils are due to less P and K adsorbed in sandy soil than silty and clayey soils [59, 60].

The animal (cattle vs. sheep; mixed vs. sheep) significantly impacted the effect sizes of SOC, TN, NO_3_^-^, K, PR, and BD (Table 1 and S3 Table). These findings indicated that the impacts of cattle grazing on SOC, TN, and available K at the 0–10 cm depth and BD at the 10–30 cm depth were significantly higher than the sheep grazing. The impacts of cattle grazing on PR at the 0–10 cm depth were significantly lower than the sheep grazing. The impacts of mixed grazing of cattle and sheep on BD, NO_3_^-^, and PR (0–10 cm) were significantly lower than the sheep grazing.

With the increase in the grazing period, the effect sizes of SOC and WC (0–10 cm) and BD (10–30 cm) significantly decreased, and the P effect size (10–30 cm) significantly increased based on the 4 mixed models (Table 1). These results indicated that with the increase in the grazing period, the grazing effects on the SOC, WC, BD reduced, and the grazing effects on the P increased.

Precipitation significantly positively impacted the effect sizes of SOC (10–30 cm), TN, P, and PR (0–10 cm) (Table 1). These results indicated that as precipitation increased, the effects of grazing on the 4 soil properties increased. Temperature significantly impacted the effect sizes of NH_4_^+^, EC, CEC, P, and PR (0–10 cm) (Table 1). These findings indicated that with the increase in temperature, the grazing effects on the NH_4_^+^, EC, and CEC increased and the P and PR decreased. Precipitation and temperature could co-vary at different spatial and temporal scales and often present hysteresis loops [61], resulting in complex effects of interactions among the grazing, precipitation, and temperature on soils. For example, grazing may interact with fluctuations in precipitation, reducing plant tillers, constraining forage production in subsequent years [62]. Precipitation and temperature can drive the directional loss of species diversity in a grassland community in semiarid regions [63]. Thus, grazing with fluctuation in precipitation and temperature could destabilize the grazing ecosystems. However, these systems can be improved if the grazing intensity is properly adjusted [64].

The impacts of interactions between the grazing and the 5 main environmental factors on the 15 soil properties are summarized in S3 Fig. The soil BD, SOC, TN, P, K, and PR were significantly impacted by more than one interaction between the grazing and the 5 environmental factors. The C: N, pH, MBC, and MBN were not impacted by any interactions (S3 Fig). The impacts of interactions between the grazing and multiple factors on soil properties have complex mechanisms, which need to be further explored. However, the grazing management (including grazing intensity) needs to be optimized by fully considering the local climate, soil texture, animals, and grazing period for maintaining soil sustainability.

### Changes in global grazing effects on soil properties over the years

This meta-analysis showed that based on the 21 mixed models, the sampling year positively impacted the effect sizes of BD, TN, pH, NO_3_^-^, K, EC, MBC, and MBN (0–10 cm) and BD, SOC, and TN (10–30 cm), but negatively impacted SOC, C: N, P, WC, NH_4_^+^, PR, and CEC (0–10 cm) and C: N, pH, and P (10–30 cm). However, all the impacts were not significant (Table 1).

The heavy and moderate grazing significantly increased the BD at the 0–10 cm depth compared to the un-grazing (Fig 3). The BD effect size was positively impacted by the year (Table 1). As a result, the soil BD (0–10 cm) under heavy and moderate grazing could have an increasing trend over the years. Similarly, the significant increases in P (0–10 cm) under the moderate grazing and PR (0–10 cm) under the heavy and moderate grazing could have an increasing trend over the years. The significant reductions in SOC (0–10 cm) by the heavy grazing, P (10–30 cm) by the moderate grazing, and WC (0–10 cm) by the heavy grazing could have a reducing trend over the years (S4 Fig). The other significant increases or reductions in the soil properties by the grazing (Fig 3) had different directions from the impacts of sampling year on their effect sizes (Table 1). For example, there was a significant reduction in SOC at the 10–30 cm depth by the heavy and moderate grazing (Fig 3), but the sampling year impact on the SOC effect size was positive (Table 1). Therefore, the trend directions of grazing effects on SOC over the years could not be determined.

The increasing trend in soil BD and reducing trend in SOC under the heavy and moderate grazing over the years (S4 Fig) indicate that the soil fertility conservation and soil water retention could have a declining trend over time [65, 66]. This signifies that the soil under the heavy and moderate grazing could degrade over time. However, considering the other 10 soil properties that could not significantly change under the heavy and moderate grazing over the years, the global soil degradation under the grazing could be low and slow over the last two decades.

### Limitations and future work

The data in this meta-analysis were only collected from English publications. All other language publications were not included. This likely results in a bias of spatial distribution of data to a certain extent worldwide. Also, the publications before 2007 were not considered in this study. Perhaps with additional data that include the period before 2007 would help to further understand the changes in the grazing effects on soil health over time. However, the lack of data could not impact the conclusions about the global grazing effects on soil properties in the last two decades. This is because our goal was to evaluate the impacts of grazing on soil properties in the last two decades due to, as stated previously, more grazing pressures on the global grasslands, compared with before 2003. Another limitation of this meta-analysis is that most studies did not report the measured data variances, which should ideally be used to weigh the effect sizes for meta-analysis. Therefore, an un-weighted meta-analysis in this study was applied, i.e., an equal variance was assumed for all studies [12, 67].

When collecting the raw data from the publications in this study, the grazing intensities (heavy, moderate, and light grazing) were defined by each publication. However, the criteria for defining the intensity were different. These criteria mainly included (i) grazing stocking rate [the number of animal unit months (AUM) supplied by one acre] based on local recommended criteria, (ii) number of animals per unit of area, (iii) grass (sward or straw) height, (iv) residue amount on cropland (e.g., corn residues for grazing on the ICLS), (v) distance to the water resource, and (vi) distance to the tree shade in grassland with trees. All the above criteria contained an inherent principle, i.e., carrying capacity of grazing land. The land carrying capacity considers both the livestock's forage needs and adequate carryover, which is the forage material that is left behind after grazing [68]. If the grazed pastures are less than their carrying capacity, the grazing in this paper refers to the heavy grazing. If the grazed pastures are close to their carrying capacity, the grazing refers to moderate, and if the grazed pastures are greater than their carrying capacity, the grazing refers to light grazing. The carrying capacity is different in various grazing lands. Therefore, it is difficult to uniformly define grazing intensity in this study. Similar situations also existed in other meta-analysis studies [19].

Most data were from the GLD and limited data came from the GLT and ICLS, thereby the land-use effects on most of the 21 effect sizes of soil property were lacking (S3 Table). Therefore, the effects of interaction between land use (ICLS, GLT, and GLD) and grazing on soil properties were not discussed. However, these effects are important for the producers and lawmakers to make decisions and policies of land-use changes (e.g., changing grassland into ICLS or adversely changing) and grazing management strategies (e.g., adjusting grazing intensity). Therefore, future work is needed to investigate the impacts of grazing in ICLS and GLD on soil properties.

## Conclusions

This meta-analysis indicates that compared to un-grazing, the heavy grazing significantly increased compaction but reduced SOC, MBC, NO_3_^-^, and soil moisture. Moderate grazing significantly increased soil compaction and soil alkalinity but reduced the SOC and TN. Light grazing significantly increased SOC and NH_4_^+^. The increase in soil compaction and the reduction in SOC, TN, C: N rate, soil moisture, and available K due to heavy grazing, compared with the un-grazing, were significantly higher than the moderate and light grazing. Impacts of cattle grazing on soil compaction, SOC, TN, and available K were significantly higher than the sheep grazing, but these impacts were lower on soil PR. Impacts of mixed grazing of cattle and sheep on BD, NO_3_^-^, and PR were significantly lower than the sheep grazing. Precipitation significantly positively impacted grazing effects on SOM, TN, available P, and PR. Temperature significantly positively impacted grazing effects on soil NH_4_^+^, EC, and CEC and negatively impacted grazing effects on available P and PR. Heavy grazing could have more detrimental impacts on soil quality than the moderate and light grazing. However, the global grazing intensities did not significantly impact most of the 15 soil properties, and the grazing impacts on the 15 soil properties had no significant changes over the last two decades. Future work is needed to further explore the mechanism of interaction between grazing and the environmental factors on soil properties, predict grazing global effects on soil quality using long-term data, and investigate grazing impacts on soils in integrated crop-livestock systems compared to grasslands.

## Supporting information

S1 Checklist PRISMA (preferred reporting items for systematic reviews and meta-analyses) checklist for this meta-analysis. (DOC)

S1 Fig Distribution of means and 90% CIs of the predicted probabilities of the 3 grazing intensities resulting in overgrazing based on the logistic models at the 0–10 cm depth. (TIF)

S2 Fig Distribution of means and 90% CIs of the predicted probabilities of the 3 grazing intensities resulting in overgrazing based on the logistic models at the 10–30 cm depth. (TIF)

S3 Fig The impacts of the interactions between the grazing and main environmental factors on the 15 soil properties. (TIF)

S4 Fig Changes in the soil properties under the heavy and moderate grazing over the years. (TIF)

S1 Table Summary information of Dataset S2 collected from the 272 publications in English from 2007 to 2017 for this meta-analysis. (DOCX)

S2 Table The percentage changes of all LS-means and CIs of the effect of grazing on the 15 soil properties. (DOCX)

S3 Table Coefficients of the independent variables as covariates in response to the grazing effect sizes (dependent variables) in the mixed models for the 0–10 and 10–30 cm depths. (DOCX)

S1 Dataset Raw data of livestock grazing effects on the 15 soil properties from 287 publications in 2007–2019. (XLSX)

S2 Dataset Compiled datasets of livestock grazing effects on the 15 soil properties from 287 publications in 2007–2019 for this meta-analysis. (XLSX)

S1 File Details of materials and methods. (DOCX)
