# Supplementary material for: A global meta-analysis of livestock grazing impacts on soil properties
Source: PLoS One. 2020 Aug 7;15(8):e0236638. doi: 10.1371/journal.pone.0236638 (PMC7413490; doi:10.1371/journal.pone.0236638)
Supplement: S1 Checklist — (DOC) [file pone.0236638.s001.doc]

| **Section/topic** | **#** | **Checklist item** | **Reported on page #** |
| --- | --- | --- | --- |
| **TITLE** | | |  |
| Title | 1 | Identify the report as a systematic review, meta-analysis, or both.  This study title is “A global meta-analysis of livestock grazing impacts on soil properties”. It is a meta-analysis. | P1 |
| **ABSTRACT** | | |  |
| Structured summary | 2 | Provide a structured summary including, as applicable: background; objectives; data sources; study eligibility criteria, participants, and interventions; study appraisal and synthesis methods; results; limitations; conclusions and implications of key findings; systematic review registration number.  The structured summary is below:  Introduction: Previous studies have showed the grazing impacts on some soil properties. However, many inconsistent results of livestock grazing intensity impacts on soil properties in different studies could mislead or neglect in addressing the soil problems resulting from the grazing. The objective of this global meta-analysis was to address the overall effects of grazing intensities (heavy, moderate, and light) on 15 soil properties using meta-analysis method.  Materials and Methods: A meta-analysis was conducted by using the mixed model method by means of PROC MIXED in SAS9.4 in this study. Effect size for this meta-analysis was defined as the natural log of the response ratio as a metric for the response of the soil property variables to grazing. Data sources were 287 papers published from 2007 to 2019 in English by searching the online database of Google Scholar using the keyword “grazing” and “soil” in the publication titles.  Results: (i) heavy grazing significantly increased the soil BD (11.3% relative) and PR (52.5%) and reduced SOC (-10.8%), WC (-10.8%), NO_3_^-^ (-23.5%), and MBC (-27.9%) at 0-10 cm depth, and reduced SOC (-22.5%) and TN (-19.9%) at 10-30 cm depth. Moderate grazing significantly increased the BD (7.5%), PR (46.0%), and P (18.9%) at 0-10 cm depth, and increased pH (4.1%) and decreased SOC (-16.4%), TN (-10.6%), and P (-23.9%) at 10-30 cm depth. Light grazing significantly increased SOC (10.8%) and NH_4_^+^ (28.7%). (ii) Soil texture, animal, precipitation, temperature, and grazing period significantly impacted the grazing effects on 4-5 soil properties, respectively. (iii) The heavy grazing showed a much higher probability (0.70) leading to overgrazing than the moderate (0.14) and light (0.10) grazing.  Conclusions: These findings indicate that, globally, compared to un-grazing, heavy grazing significantly increased soil compaction and reduced SOC, NO3-, and soil moisture. Moderate grazing significantly increased soil compaction and alkalinity and reduced SOC and TN. Light grazing significantly increased SOC and NH4+. Cattle grazing impacts on soil compaction, SOC, TN, and available K were higher than sheep grazing, but lower for PR. Climate significantly impacted grazing effects on SOM, TN, available P, NH4+, EC, CEC, and PR. Heavy grazing can be more detrimental to soil quality based on BD, SOC, TN, C: N, WC, and K than moderate and light grazing. However, global grazing intensities did not significantly impact most of the 15 soil properties, and the grazing effects on them had insignificant changes over the years. | P2 |
| **INTRODUCTION** | | |  |
| Rationale | 3 | Describe the rationale for the review in the context of what is already known.  Previous studies have showed the grazing impacts on some soil properties. However, many inconsistent results of livestock grazing intensity impacts on soil properties in different studies could mislead or neglect in addressing the soil problems resulting from the grazing. This study uses 15 various soil properties all together across a wide range of environments in the world those were not studied before. This study provides a comprehensive understanding in grazing intensity impacts on soils from global, temporal, and environmental perspectives, and how these understandings impact soils to manage the grazing more appropriately, especially where it is advocated as a tool to manage the conservation of soils in the livestock-pasture system. | P3-4 |
| Objectives | 4 | Provide an explicit statement of questions being addressed with reference to participants, interventions, comparisons, outcomes, and study design (PICOS).  Utilizing comprehensive meta-analytical approach based on publications from 2007 to 2019, the objective of this study was to address the overall effects of global grazing intensities on 15 soil properties. | P4 |
| **METHODS** | | |  |
| Protocol and registration | 5 | Indicate if a review protocol exists, if and where it can be accessed (e.g., Web address), and, if available, provide registration information including registration number.  There is not a review protocol exists (also no registration number). However, the meta-analysis method in this study was described in the manuscript and S1 File in the supporting information. |  |
| Eligibility criteria | 6 | Specify study characteristics (e.g., PICOS, length of follow-up) and report characteristics (e.g., years considered, language, publication status) used as criteria for eligibility, giving rationale.  We selected the papers published from 2007 to 2019 in English for this meta-analysis. This is because there was not a meta-analysis for evaluating the global effects of livestock grazing on 15 soil properties using the recent decade publications, and these effects are important for comprehensively understanding the grazing intensity impacts on soils from the global, temporal, and environmental perspectives. Furthermore, although the total annual meat and milk from cattle, sheep, and goats in the world increased from 1961 to 2018, the total annual meat and milk production increased at a faster rate (greater than 23 million tons and 180 million tons, respectively) since 2003. Additionally, the global annual area of permanent meadows and pastures decreased by less than 3.4 million ha since 2003. Increased meat and milk production and decreased permanent grasslands indicate increased grazing pressure on the livestock grazing lands since 2003. The increased grazing pressure likely led to increases in soil property changes. The studies published since 2007 could cover the data of the grazing effects on the soil properties since 2003. | P3-4 |
| Information sources | 7 | Describe all information sources (e.g., databases with dates of coverage, contact with study authors to identify additional studies) in the search and date last searched.  Data were from these identified publications that were searched in the online database of Google Scholar from Jan. 1, 2007 to Dec. 31, 2019. | P5 |
| Search | 8 | Present full electronic search strategy for at least one database, including any limits used, such that it could be repeated.  We searched the online database of Google Scholar using the keyword “grazing” and “soil” in the titles and obtained the information of 1,260 publications. | P5 |
| Study selection | 9 | State the process for selecting studies (i.e., screening, eligibility, included in systematic review, and, if applicable, included in the meta-analysis).  We checked each study of above 1,260 publications and identified the useful publications (368 publications) for this meta-analysis after removing those that did not match the objectives of this study and did not find their full-texts or useful data from them. Finally, the 287 publications were selected for this meta-analysis after removing those with ineligibility data based on this study criteria. | P5 |
| Data collection process | 10 | Describe method of data extraction from reports (e.g., piloted forms, independently, in duplicate) and any processes for obtaining and confirming data from investigators.  The data were collected from the tables or extracted from the digitized graphs using WebPlotDigitizer software (Version 3.8 for Desktop) from these publications after carefully checking them. Some missing information such as coordinate, altitude, temperature, and precipitation in some publications were collected from the Websites based on the locations provided in these studies. | P5 |
| Data items | 11 | List and define all variables for which data were sought (e.g., PICOS, funding sources) and any assumptions and simplifications made.  The soil properties include 15 properties: soil bulk density (BD), penetration resistance (PR), soil organic carbon (SOC), total nitrogen (TN), C: N ratio, ammonium (NH_4_^+^), nitrate (NO_3_^-^), microbial biomass carbon (MBC), microbial biomass nitrogen (MBN), available phosphorus (P), and available potassium (K), cation exchange capacity (CEC), pH, electrical conductivity (EC), and water content (WC). Site information includes grazing intensity, overgrazing, grazed animals, grazing period (year), land-use, sampling year and soil depth, latitude, longitude, altitude, country, soil texture, mean annual precipitation, and mean daily temperature.  The data collection from publications followed the criteria and assumptions. (i) Soil property data under various grazing intensities (≥3) and the un-grazing from different field sites at 0-30 cm depth were collected. Because grazing data cannot be compared to un-grazing at the same time, this study excluded temporal comparisons data (before and after grazing) of the same site. (ii) Data at sites grazed by domestic animals such as cattle (Bos Taurus L.), sheep (Ovis aries L.) (including goat and deer), or their mixture were selected. (iii) This study defined non-adjacent sites to have a distance greater than 46 km (an approximate distance of 25’ in latitude range). Non-adjacent site data were considered independent and included in the database. Adjacent site data were only limited to one site to avoid dependent data with different sites. (iv) Data collected within a year for each site were averaged [12]. Data collected over multiple years could be time-dependent (e.g., autocorrelation). As a result, only the data from the most recent year were included. (v) Site information including grazing intensity, overgrazing, grazing animals, grazing period, land-use, sampling year, soil depth, latitude, longitude, altitude, country, soil texture, mean annual precipitation, and mean daily temperature were all collected. Missing information such as latitude, longitude, altitude, temperature, and precipitation in some publications were retrieved from the websites (https://www.ncdc.noaa.gov/cdo-web/datasets; http://www.worldclimate.com/; https://www.freemaptools.com/elevation-finder.htm; https://www.google.com/maps/) based on the information of study locations. The grazing intensity level (heavy, moderate, or light) and overgrazing (or non-overgrazing) were defined based on the authors’ original studies. Due to the wide range of livestock types and units used in different studies, the intensity has various stocking rates. This study also defined three types of the lands: grassland (GLD), grassland with trees (GLT) (including forests for grazing), and integrated crop-livestock system (ICLS). | P4-6 |
| Risk of bias in individual studies | 12 | Describe methods used for assessing risk of bias of individual studies (including specification of whether this was done at the study or outcome level), and how this information is to be used in any data synthesis.  The data in different studies were regarded as independent. Most studies have one experiment site. For the study with more than one site, if the sites were adjacent, only one dataset from the sites was selected to assure the data are independent. If the sites were not adjacent (we supposed the distance among the sites >46 km, an approximate distance for a latitude range of 25', in the study), the data from different sites were regarded as independent and included in the database. Data from one site but in different times during a year were averaged to one value. Data from the same site in different years could be time-dependent (e.g., autocorrelation), only the data from the most recent year were included and the data from the other years at the same site were excluded. This indicate that the possible time-correlation of data in our database for this meta-analysis was deleted. Therefore, there are not the time-correlation data for this meta-analysis. | P5 |
| Summary measures | 13 | State the principal summary measures (e.g., risk ratio, difference in means).  Effect size (ES) for this meta-analysis was defined as the natural log of the response ratio as a metric for the response of the soil property variables to grazing. For a given variable X (soil property), the X effect size was calculated based on the equation:  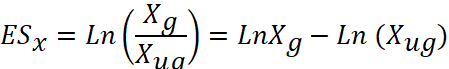  where *ES_x_* is the effect size of X; *X_g_* is the value of X in the grazing group; *X_ug_* is the value of X in the un-grazing group. This study examined a total of 15 soil properties. | P7 |
| Synthesis of results | 14 | Describe the methods of handling data and combining results of studies, if done, including measures of consistency (e.g., I^2^) for each meta-analysis.   1. In this study, the most publications for collecting the meta-analysis data did not report the measured data variances (or standard deviation), which should ideally be used to weight the effect sizes for meta-analysis. Therefore, an un-weighted meta-analysis in this study was applied, i.e., an equal variance was assumed for all studies (*The previous two meta-analysis studies also did the same thing. (i) Guo LB, Gifford R. Soil carbon stocks and land use change: a meta analysis. Global Change Biol. 2002;8(4):345-60. (ii) Wang X, McConkey BG, VandenBygaart A, Fan J, Iwaasa A, Schellenberg M. Grazing improves C and N cycling in the Northern Great Plains: A meta-analysis. Sci Rep. 2016;6:33190*). Since measured data variances for most of the publications are unavailable, the weight could not be calculated, the Cochran’s Q and I^2^ cannot be calculated. However, based on our assumption (an equal variance was assumed for all studies), we tried using 1 as weight for all effect sizes to calculate Cochran’s Q and I^2^ of all effect sizes of soil properties in S2 Dataset. We found that all I^2^ for the 15 effect sizes of soil properties were negative, indicating that the heterogeneity of the 15 effect sizes was very small. 2. Mixed model method was used for conducting this meta-analysis using PROC MIXED in SAS9.4 in this study. The mixed model method is appropriate for analyzing differences between groups of experiments when the groups are not expected to be internally homogeneous. The mixed models can address the limitation of using randomization tests (resampling method) that cannot separate the two sources of variance (within-study sampling error and between-study variation in true effects). The 21 mixed models were built using the 15 soil property effect sizes as dependent variables at the 0-10 and 10-30 cm depths. The grazing intensity as main effect and the soil texture, land-use, grazing animal, latitude, altitude, precipitation, temperature, grazing period, and sampling year (the 9 variables were regarded as covariates for adjusting the mean effect sizes among the 3 grazing intensities) were taken as the fixed effects, and the study ID was as the random effect in the mixed models. The least square means (LS-means) of soil property effect sizes and their confidence intervals (CIs) of the 3 grazing intensities were estimated in the mixed models based on the SAS algorithm, by which the LS means were adjusted by covariates in the mixed models and were less sensitive to missing data. The estimated LS-means and CIs of the soil property effect sizes indicate the grazing intensity effects on the soil properties. The LS-means and CIs were reported as the percentage change estimated by (*e^ES^* − 1) × 100%. | P8-9 |

Page 1 of 2

| **Section/topic** | **#** | **Checklist item** | **Reported on page #** |
| --- | --- | --- | --- |
| Risk of bias across studies | 15 | Specify any assessment of risk of bias that may affect the cumulative evidence (e.g., publication bias, selective reporting within studies).  The publication bias indicate that the publication of studies depends on the direction and statistical significance of the results. Publication bias generally leads to effect sizes being overestimated and the dissemination of false-positive results. The presence of publication bias is present in various research fields. Especially, in the psychology and psychiatry, their literature is statistically significant in approximately 90% of the cases. This is because most insignificant results (or significantly negative results) of medicine studies cannot published because they could not use to treat diseases. However, in the soil research fields, the publication bias is not prominent because the soil studies are different from the medicine studies: all the positive and negative results of soil studies (no matter whether they are significantly different or not) can be published. In this study, based on our database compiled (S2 Dataset), there are a total of 2788 effect sizes, in which the number of positive, zero, and negative effect sizes are 1328, 64, and 1396, respectively. The negative effect sizes are slightly more than the positive effect sizes. Therefore, this study has small publication bias. |  |
| Additional analyses | 16 | Describe methods of additional analyses (e.g., sensitivity or subgroup analyses, meta-regression), if done, indicating which were pre-specified.  (1) For other independent character variables [not include “grazing intensity”, which was for estimating the least square means (LS-means) of soil property effect sizes and their confidence intervals] in the mixed models (see Item 14), we cannot use their LS means to explain their effects on soil properties because the dependent variables were only the grazing effect sizes. However, the coefficients and p-values of the variables of fixed effects in the mixed models can be used to interpret their impacts on soil property effect sizes of the grazing, namely, effects of interaction between these variables and the grazing on the soil properties.  (2) The binomial logistic models were built to predict the probabilities of the 3 grazing intensities resulting in the overgrazing using SAS9.4. | P8-10 |
| **RESULTS** | | |  |
| Study selection | 17 | Given numbers of studies screened, assessed for eligibility, and included in the review, with reasons for exclusions at each stage, ideally with a flow diagram.  The 287 publications were finally selected for collecting data for this meta-analysis. The detail is presented in Fig 1 (a flow diagram). | P5 and Fig. 1 |
| Study characteristics | 18 | For each study, present characteristics for which data were extracted (e.g., study size, PICOS, follow-up period) and provide the citations.  (1) Characteristics and citation of each study was described in S1 Dataset in Supporting Information.  (2) Summary information of the compiled data from the 287 publications for this meta-analysis was described in S1 Table and S2 Dataset in Supporting Information. | S1 Dataset, S1 Table and S2 Dataset in Supporting Information |
| Risk of bias within studies | 19 | Present data on risk of bias of each study and, if available, any outcome level assessment (see item 12).  The studies with possible bias within them have been avoided when we selected these publications based on our criteria for identifying the publications (see item 12). Therefore, the data in different studies were independent in this manuscript. |  |
| Results of individual studies | 20 | For all outcomes considered (benefits or harms), present, for each study: (a) simple summary data for each intervention group (b) effect estimates and confidence intervals, ideally with a forest plot.  Results (effect sizes) of each study was described in S2 Dataset (the column GES represents effect size) in Supporting Information. The effect size was defined in item 13. The forest plot for effect estimates and confidence intervals is presented in Fig. 3. | S2 Dataset (the column GES represents effect size) and Fig. 3. |
| Synthesis of results | 21 | Present results of each meta-analysis done, including confidence intervals and measures of consistency.  The results in this meta-analysis study (including confidence intervals and measures of consistency) are presented in Fig 3 (a forest plot) and S2 Table. The highlights are that the heavy grazing significantly increased the soil BD (11.3% relative) and PR (52.5%) and reduced SOC (-10.8%), WC (-10.8%), NO_3_^-^ (-23.5%), and MBC (-27.9%) at 0-10 cm depth, and reduced SOC (-22.5%) and TN (-19.9%) at 10-30 cm depth. Moderate grazing significantly increased the BD (7.5%), PR (46.0%), and P (18.9%) at 0-10 cm depth, and increased pH (4.1%) and decreased SOC (-16.4%), TN (-10.6%), and P (-23.9%) at 10-30 cm depth. Light grazing significantly increased SOC (10.8%) and NH_4_^+^ (28.7%). | P10-11 |
| Risk of bias across studies | 22 | Present results of any assessment of risk of bias across studies (see Item 15).   1. In this study, based on our database compiled (S2 Dataset), there are a total of 2788 effect sizes, in which the number of positive, zero, and negative effect sizes are 1328, 64, and 1396, respectively. The negative effect sizes are slightly more than the positive effect sizes. Therefore, this study has small publication bias. 2. The data were from the publications that were independent studies each other. Moreover, when building the 21 models, we used 3 methods to overcome these risks of the models (see Item 15), thereby, the models are “good”. |  |
| Additional analysis | 23 | Give results of additional analyses, if done (e.g., sensitivity or subgroup analyses, meta-regression [see Item 16]).  (1) In response to (1) in Item 16, the results of additional analyses are presented in Table 1 and S3 Table.  (2) In response to (2) in Item 16, the results of additional analyses are presented in Table 2 and S1 and S2 Figs. | P12-15 |
| **DISCUSSION** | | |  |
| Summary of evidence | 24 | Summarize the main findings including the strength of evidence for each main outcome; consider their relevance to key groups (e.g., healthcare providers, users, and policy makers).  (1) Global impacts of livestock grazing on soil properties. The summary of evidence is presented in the above mechanisms of grazing impacts on 15 soil properties (Fig 4). The details were described in section 1 in the discussion in manuscript.  (2) Interactions between grazing and environmental factors and the impacts on soil properties. We explained main findings with evidence. The details were described in section 2 in the discussion in manuscript.  (3) Effects of grazing on soils over the years. We explained the findings with evidence. The details were described in section 3 in the discussion in manuscript. | P15-20 |
| Limitations | 25 | Discuss limitations at study and outcome level (e.g., risk of bias), and at review-level (e.g., incomplete retrieval of identified research, reporting bias).  The 4 limitations were discussed in this study. The details were described in page 20-21. | P21-22 |
| Conclusions | 26 | Provide a general interpretation of the results in the context of other evidence, and implications for future research.  This meta-analysis indicates that compared to un-grazing, the heavy grazing significantly increased compaction but reduced SOC, MBC, NO3-, and soil moisture. Moderate grazing significantly increased soil compaction and soil alkalinity but reduced the SOC and TN. Light grazing significantly increased SOC and NH4+. The increase in soil compaction and the reduction in SOC, TN, C: N rate, soil moisture, and available K due to heavy grazing, compared with the un-grazing, were significantly higher than the moderate and light grazing. Impacts of cattle grazing on soil compaction, SOC, TN, and available K were significantly higher than the sheep grazing, but these impacts were lower on soil PR. Impacts of mixed grazing of cattle and sheep on BD, NO3-, and PR were significantly lower than the sheep grazing. Precipitation significantly positively impacted grazing effects on SOM, TN, available P, and PR. Temperature significantly positively impacted grazing effects on soil NH4+, EC, and CEC and negatively impacted grazing effects on available P and PR. Heavy grazing could have more detrimental impacts on soil quality than the moderate and light grazing. However, the global grazing intensities did not significantly impact most of the 15 soil properties, and the grazing impacts on the 15 soil properties had no significant change over the last two decades. Future work is needed to further explore the mechanism of interaction between grazing and the environmental factors on soil properties, predict grazing global effects on soil quality using long-term data, and investigate grazing impacts on soils in integrated crop-livestock systems compared to grasslands. | P22-23 |
| **FUNDING** | | |  |
| Funding | 27 | Describe sources of funding for the systematic review and other support (e.g., supply of data); role of funders for the systematic review.  This review is a contribution from the United States Department of Agriculture-NIFA, Coordinated Agricultural Projects (CAP) (Award Number 2016-68004-24768) and the project entitled “Back to the Future: Enhancing food security and farm production with integrated crop-livestock production systems”. | P23 |

*From:*  Moher D, Liberati A, Tetzlaff J, Altman DG, The PRISMA Group (2009). Preferred Reporting Items for Systematic Reviews and Meta-Analyses: The PRISMA Statement. PLoS Med 6(7): e1000097. doi:10.1371/journal.pmed1000097

For more information, visit: **www.prisma-statement.org**. Page 2 of 2
