# Supplementary figures and images for: A global meta-analysis of livestock grazing impacts on soil properties
Source: PLoS One. 2020 Aug 7;15(8):e0236638. doi: 10.1371/journal.pone.0236638 (PMC7413490; doi:10.1371/journal.pone.0236638)

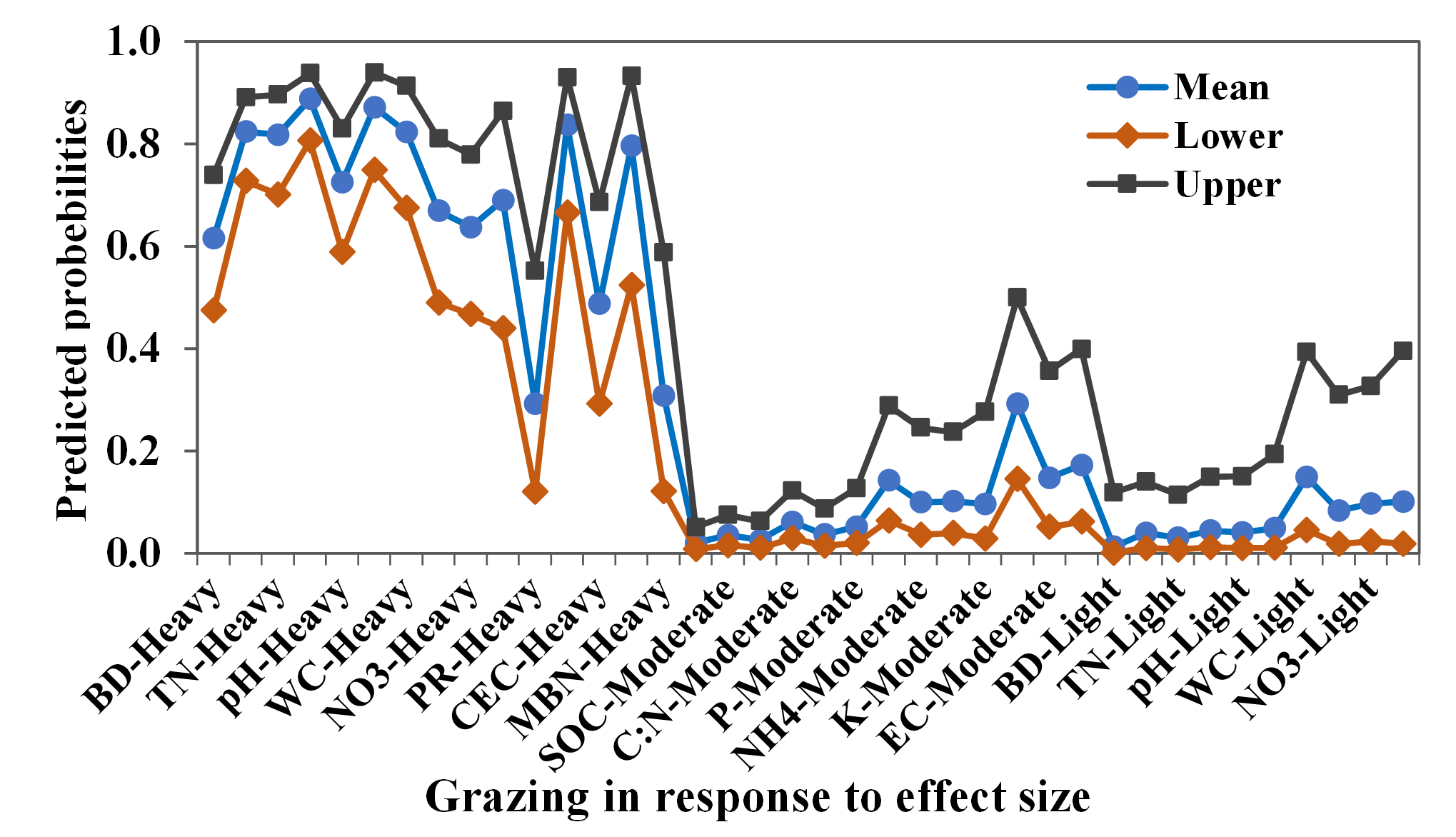

Supplement: S1 Fig — (TIF) [file pone.0236638.s002.tif]

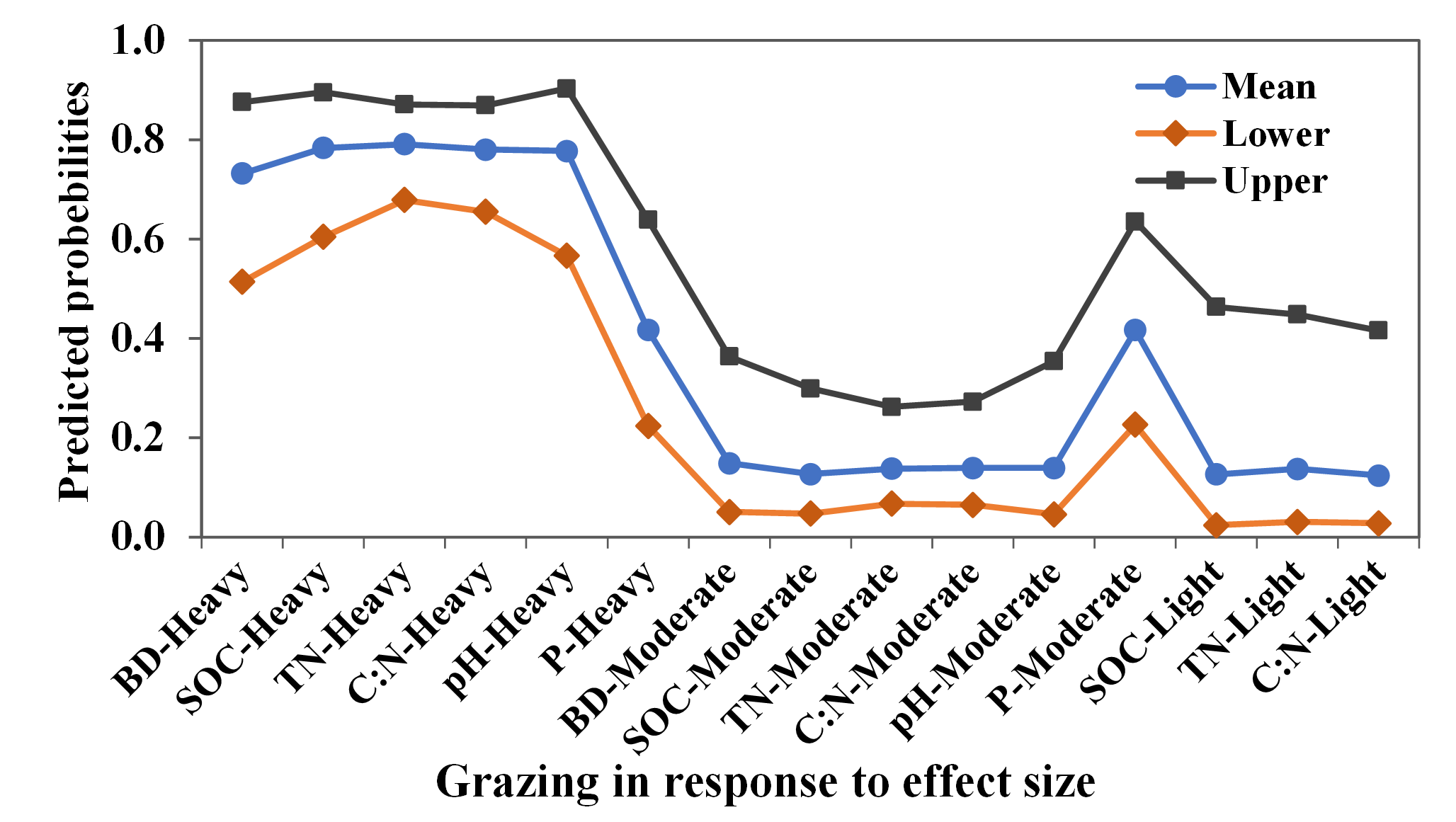

Supplement: S2 Fig — (TIF) [file pone.0236638.s003.tif]

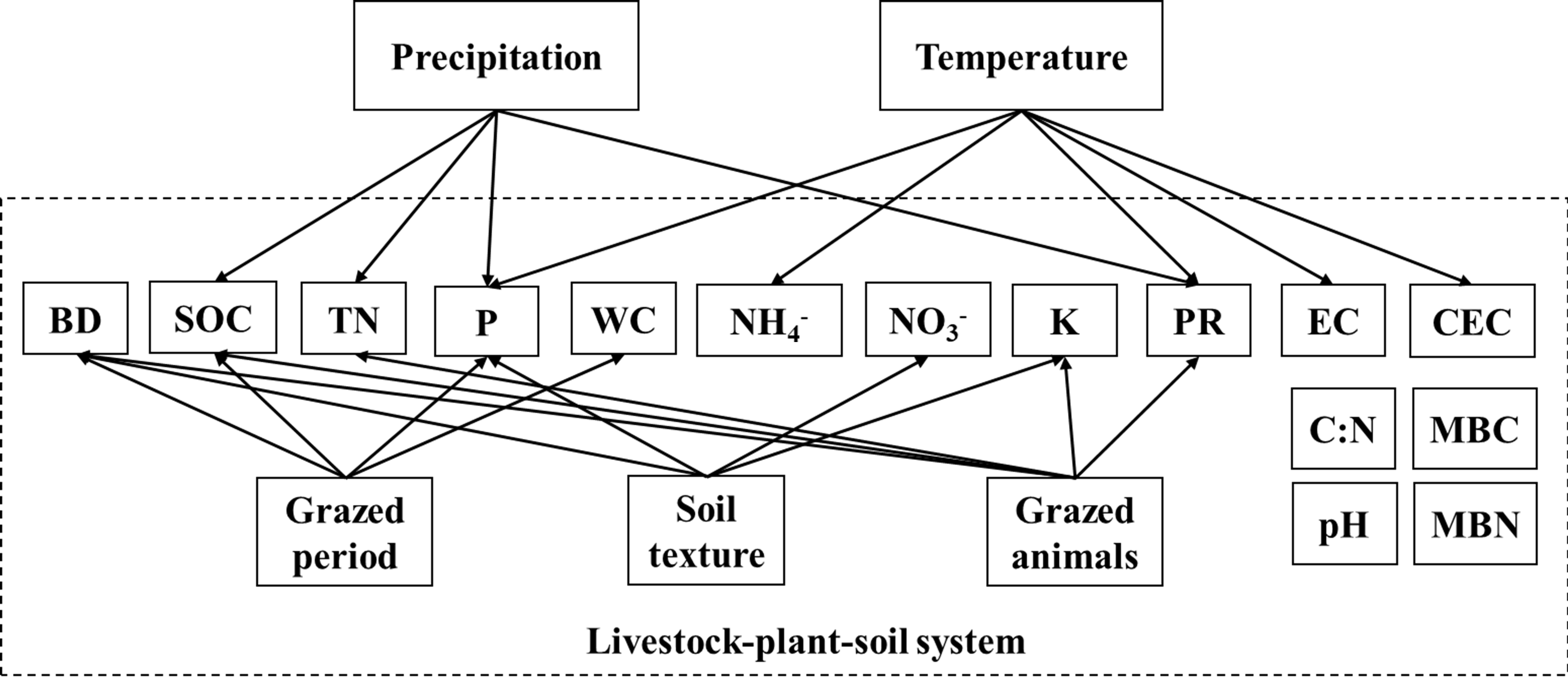

Supplement: S3 Fig — (TIF) [file pone.0236638.s004.tif]

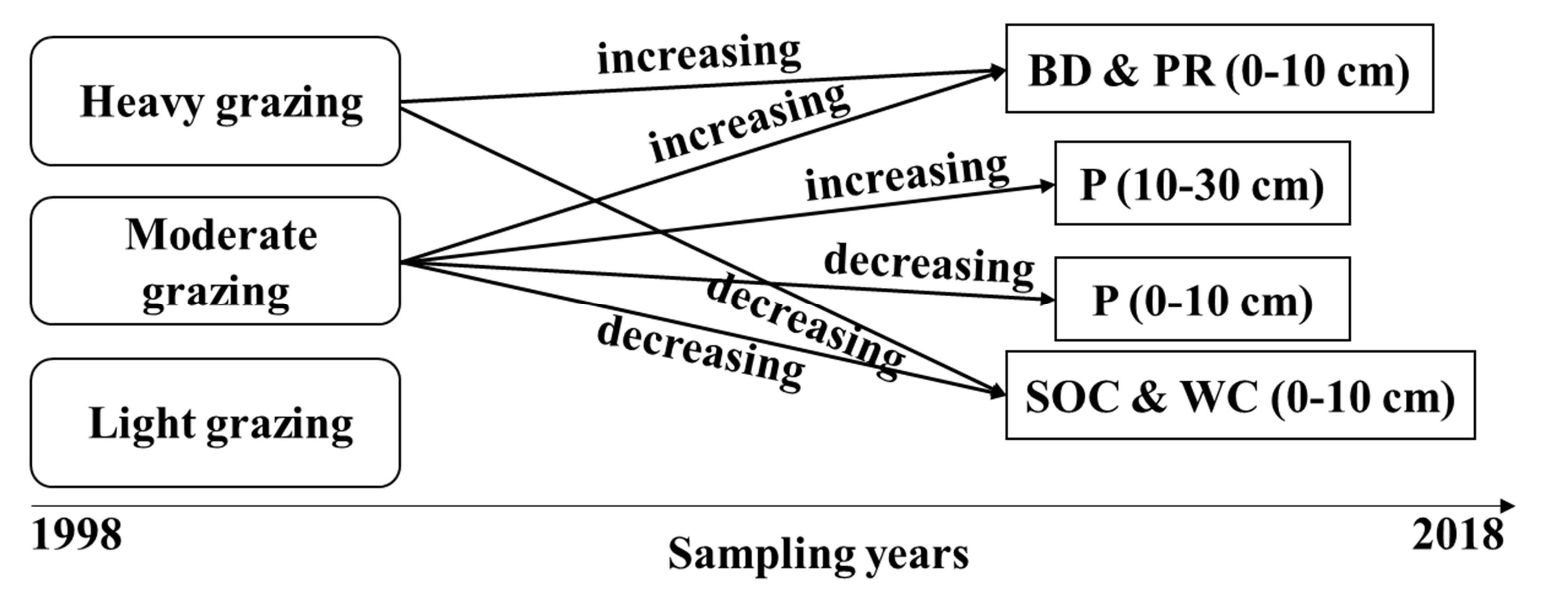

Supplement: S4 Fig — (TIF) [file pone.0236638.s005.tif]
