# Supplementary material for: A global meta-analysis of livestock grazing impacts on soil properties
Source: PLoS One. 2020 Aug 7;15(8):e0236638. doi: 10.1371/journal.pone.0236638 (PMC7413490; doi:10.1371/journal.pone.0236638)
Supplement: S1 Table — (DOCX) [file pone.0236638.s006.docx]

**S1 Table.** Summary information of S2 Dataset collected from the 289 publications in English from 2007 to 2019 for this meta-analysis.

| **Soil variables**^a^ | **Lines of grazing effect sizes** | | | **Total papers#** | **Sampling years** |
| --- | --- | --- | --- | --- | --- |
|  | **0-10cm** | **10-30cm** | **Total** |  |  |
| BD | 268 | 83 | 351 | 143 | 1999-2017 |
| SOC | 359 | 110 | 469 | 195 | 1998-2018 |
| TN | 302 | 109 | 411 | 163 | 1999-2018 |
| C: N | 275 | 95 | 370 | 149 | 1999-2018 |
| pH | 259 | 51 | 310 | 139 | 2000-2017 |
| P | 162 | 44 | 206 | 86 | 2000-2017 |
| WC | 154 | - | 154 | 81 | 1999-2017 |
| NH_4_^+^ | 75 | - | 75 | 42 | 1999-2015 |
| NO_3_^-^ | 92 | - | 92 | 49 | 1999-2015 |
| K | 74 | - | 74 | 38 | 2000-2017 |
| PR | 54 | - | 54 | 28 | 1999-2016 |
| EC | 64 | - | 64 | 38 | 2003-2015 |
| CEC | 51 | - | 51 | 30 | 2003-2017 |
| MBC | 73 | - | 73 | 43 | 1999-2018 |
| MBN | 34 | - | 34 | 23 | 2005-2018 |
| ***Summary*** | *2296* | *492* | *2788* | *287 papers* | *12-20 years* |

^a^ In addition to the 15 soil property variables, our dataset S2 also contained 14 site information variables: grazing intensity, overgrazing, soil depth, soil texture, land-use, grazing animal, country, latitude, longitude, altitude, precipitation, temperature, grazing period, and sampling year, and 5 publication information variables: Author, published year, country, abstract, reference.
