# Supplementary material for: A global meta-analysis of livestock grazing impacts on soil properties
Source: PLoS One. 2020 Aug 7;15(8):e0236638. doi: 10.1371/journal.pone.0236638 (PMC7413490; doi:10.1371/journal.pone.0236638)
Supplement: S2 Table — (DOCX) [file pone.0236638.s007.docx]

**S2 Table**. The percentage changes of all LS-means and CIs of effect of grazing on the 15 soil properties.

| **Soil properties** | **depth (cm)** | **Heavy grazing** | | | **Moderate grazing** | | | **Light grazing** | | |
| --- | --- | --- | --- | --- | --- | --- | --- | --- | --- | --- |
|  |  | **LS-mean** | **Lower** | **Upper** | **LS-mean** | **Lower** | **Upper** | **LS-mean** | **Lower** | **Upper** |
| BD | 0-10 | 11.3 | 8.9 | 13.7 | 7.5 | 5.2 | 9.9 | -0.4 | -3.4 | 2.8 |
| BD | 10-30 | 2.4 | 5.5 | -0.5 | -0.2 | 3.1 | -3.2 | 0.0 | 2.8 | -2.7 |
| SOC | 0-10 | -10.8 | -17.7 | -3.8 | 1.9 | -5.2 | 9.2 | 10.9 | 1.1 | 20.8 |
| SOC | 10-30 | -22.5 | -33.9 | -10.2 | -16.4 | -28.7 | -3.0 | -9.9 | -24.5 | 6.0 |
| TN | 0-10 | -4.7 | -12.0 | 3.0 | 1.3 | -6.6 | 9.5 | 8.6 | -1.5 | 19.3 |
| TN | 10-30 | -19.9 | -27.2 | -12.2 | -10.6 | -18.9 | -1.9 | -8.3 | -20.2 | 4.5 |
| C:N | 0-10 | -3.6 | -8.1 | 0.9 | -0.9 | -5.4 | 3.8 | 1.4 | -4.3 | 7.2 |
| C:N | 10-30 | -1.8 | -8.3 | 4.9 | 1.5 | -5.6 | 9.0 | 3.0 | -7.2 | 13.8 |
| pH | 0-10 | -0.1 | -1.5 | 1.4 | 0.2 | -1.3 | 1.7 | -0.3 | -2.2 | 1.6 |
| pH | 10-30 | 1.3 | -1.9 | 4.5 | 4.1 | 0.9 | 7.4 | - | - | - |
| P | 0-10 | 16.0 | -0.8 | 35.7 | 18.9 | 2.3 | 38.1 | 19.1 | -1.9 | 44.5 |
| P | 10-30 | -20.0 | -37.4 | 2.2 | -23.9 | -40.9 | -2.1 | - | - | - |
| WC | 0-10 | -10.8 | -18.4 | -2.5 | 2.6 | -6.3 | 12.3 | 11.6 | -0.1 | 24.8 |
| NH4 | 0-10 | 14.6 | -2.6 | 35.0 | 12.0 | -4.7 | 31.6 | 28.7 | 3.6 | 60.0 |
| NO3 | 0-10 | -23.5 | -36.4 | -6.7 | -14.9 | -30.3 | 5.3 | -19.3 | -36.4 | 4.6 |
| K | 0-10 | -4.3 | -19.5 | 12.5 | -5.1 | -20.2 | 11.8 | 15.8 | -4.2 | 38.5 |
| PR | 0-10 | 52.5 | 17.9 | 97.4 | 46.0 | 16.0 | 83.7 | - | - | - |
| EC | 0-10 | 0.4 | -21.6 | 26.6 | -5.0 | -27.8 | 22.8 | - | - | - |
| CEC | 0-10 | -14.5 | -27.9 | 1.4 | -12.7 | -26.3 | 3.4 | - | - | - |
| MBC | 0-10 | -27.9 | -45.5 | -4.6 | -6.2 | -27.8 | 21.8 | -10.0 | -35.6 | 25.7 |
| MBN | 0-10 | 2.7 | -25.5 | 41.4 | 1.9 | -23.4 | 35.6 | - | - | - |
