# Supplementary material for: A global meta-analysis of livestock grazing impacts on soil properties
Source: PLoS One. 2020 Aug 7;15(8):e0236638. doi: 10.1371/journal.pone.0236638 (PMC7413490; doi:10.1371/journal.pone.0236638)
Supplement: S3 Table — (DOCX) [file pone.0236638.s008.docx]

**S3 Table.** The coefficients of the soil texture (Sandy vs others), land use (GLD vs ICLS and GLT vs ICLS), animal (Cattle vs. sheep and Mix vs Sheep), grazed period (Period), latitude (Lati), Altitude, precipitation (Prcp), temperature (Temp), and sampling year (Year) in the 21 mixed models for the 0-10 and 10-30 cm depths.

| **Effect sizes** | **Depth (cm)** | **Sandy vs Others**^a^ | **GLD vs ICLS**^a^ | **GLT vs ICLS**^a^ | **Cattle vs Sheep** | **Mix vs Sheep**^a^ | **Period (1/100)** | **Lati (1/1000)** | **Altitude (1/10000)** | **Prcp (1/10000)** | **Temp (1/100)** | **Year (1/100)** |
| --- | --- | --- | --- | --- | --- | --- | --- | --- | --- | --- | --- | --- |
|  |  |  |  |  |  |  |  |  |  |  |  |  |
| BD | 0-10 | -0.019* | 0.022* | 0.031* | -0.004 | -0.022* | 0.043 | 0.168 | 0.019 | 0.16 | -0.069 | 0.044 |
| SOC | 0-10 | -14.45 | 11.82 | 75.90* | 65.83* | 32.08 | -125.4* | 309.5 | -28 | 52.1 | -4.694 | -18.2 |
| TN | 0-10 | -0.015 | -4.089 | 4.934 | 5.567* | 2.696 | -6.699 | -52.54 | 8.84 | 76.98* | -13.57 | 44.14 |
| C:N | 0-10 | -0.183 | - | - | 0.237 | 0.152 | -0.385 | 2.316 | -1.800* | 0.39 | -1.105 | -3.544 |
| pH | 0-10 | -0.002 | 0.01 | -0.015 | -0.013 | -0.005 | 0.03 | -0.04 | -0.100* | 0.033 | -0.02 | 0.054 |
| P | 0-10 | 0.037 | - | - | 0.111 | 0.104 | -0.439 | -4.72 | -0.6 | 3.990* | -2.614* | -0.759 |
| WC | 0-10 | 0.021 | - | - | 0.069 | 0.088 | -0.472* | 0.846 | -0.2 | 0.15 | 0.296 | -0.553 |
| NH_4_^+^ | 0-10 | -0.138 | - | - | 0.236 | - | 0.184 | 9.564 | -0.018 | 2.36 | 2.963* | -0.553 |
| NO_3_^-^ | 0-10 | -0.141* | - | - | -0.106 | -0.275* | 0.158 | 1.566 | 0.29 | 1.19 | 0.312 | 0.554 |
| K | 0-10 | 2.156* | - | - | 2.266* | 1.177 | -0.435 | 8.923 | -1 | 22.94 | -2.698 | 6.288 |
| PR | 0-10 | 0.031 | 0.155 | 0.042 | -0.781* | -0.929* | 0.982 | 1.929 | 0.97 | 6.520* | -4.222* | -4.955 |
| EC | 0-10 | -3.177 | - | - | 1.855 | -3.375 | -10.6 | 112.8* | -18.9 | 4.14 | 31.00* | 37.53 |
| CEC | 0-10 | 0.065 | - | - | - | - | -0.45 | -2.13 | 0.35 | 2.17 | 2.126* | -1.134 |
| MBC | 0-10 | 0.046 | - | - | 0.026 | - | 0.272 | 2.013 | -0.3 | -1.9 | 1.531 | 1.251 |
| MBN | 0-10 | 0.346 | - | - | -0.202 | - | 1.705 | -3.16 | 1.3 | 1.84 | 0.534 | 0.047 |
| BD | 10-30 | -0.011 | 0.032* | - | 0.031* | 0.006 | -0.080* | 0.057 | 0.082 | -0.2 | 0.05 | 0.069 |
| SOC | 10-30 | -0.114 | 6.157 | - | 1.454 | 1.017 | -6.916 | 37.99 | -54.7 | 116.5* | -9.453 | 2.203 |
| TN | 10-30 | 1.884 | - | - | 1.759 | 2.918 | 17.56 | 53.58 | -23.5 | 75.97 | 40.74 | 1.892 |
| C:N | 10-30 | 0.087 | - | - | -0.241 | -0.106 | 0.163 | -3.74 | -2.8 | 2.12 | -3.03 | -3.429 |
| pH | 10-30 | -0.002 | - | - | -0.047 | 0.007 | 0.035 | -0.33 | -0.097 | 0.034 | -0.114 | -0.297 |
| P | 10-30 | 0.753* | - | - | 0.106 | 0.328 | 2.428* | -7.18 | 0.3 | -0.1 | -0.881 | -5.504 |

^a^ Others, silty and clayey soils; GLD, grassland; ICLS, integrated crop-livestock system; GLT, grassland with trees; Mix, mixed animals (cattle and sheep).

* indicates that the independent variable significantly impacted the effect size of soil property in the mixed model (P<0.10). However, the coefficients of independent variables cannot be used to interpret how many percentages they can increase or reduce their effect sizes of soil properties because most of the dependent variables (i.e., effect sizes) were transformed using different algorithms based on Box-Cox method when these best models were built.
