# Supplementary material for: A global meta-analysis of livestock grazing impacts on soil properties
Source: PLoS One. 2020 Aug 7;15(8):e0236638. doi: 10.1371/journal.pone.0236638 (PMC7413490; doi:10.1371/journal.pone.0236638)
Supplement: S1 File — (DOCX) [file pone.0236638.s011.docx]

**S1 File. Details of materials and methods**

**Data compilation**

287 publications (the references are in S1 and S2 Datasets) were selected for collecting data, which were summarized as S1 Dataset and then compiled as S2 Dataset for our meta-analysis after checking the 1,260 published papers in English from 2007 to 2019 by searching the online database of Google Scholar using the keyword “grazing” and “soil” in the paper titles. The sampling years involved in 1998-2018 and sampling sites distributed in 52 countries.

Raw data collection from the searched publications followed the criteria and methods. (i) The soil property data under the grazing treatments at field sites were only collected. The studies that made temporal comparisons of the same site before and after grazing were excluded. (ii) Experiments grazed by domestic animals such as cattle, sheep (or goats), or their mixture were selected. Some experiments grazed by deer were also selected as sheep type because their weights and activities are similar. (iii) Sites information such as grazing intensity, overgrazing, soil depth, soil texture, land-use, grazed animal, country, latitude, longitude, altitude, mean annual precipitation (MAP), mean daily temperature (MDT), grazing period, and sampling year were also collected. The measurements of the above variables in the grazing treatments were required to be performed at the same temporal and special scales. If MAP and MDT could not be found in a primary study, we used the weather data that were approximated from the nearest weather station in the USA and Canada and collected using the online database of Climate Data Online (CDO) and National Climatic Data Center (NCDC) (https://www.ncdc.noaa.gov/cdo-web/datasets). If the site was out of the USA and Canada, the weather data were approximated from the nearest city and collected using the online database of World Climate (http://www.worldclimate.com/). The altitude data of some cases without elevation information were collected from the online database of Free Map Tools (https://www.freemaptools.com/elevation-finder.htm). Data on latitude and longitude at some sites without geographical coordinates were approximately obtained from Google Map (https://www.google.com/maps/) in terms of the information of sites in the studies. Data for overgrazing and non-overgrazing in some studies were indirectly determined in terms of the results or conclusions of the studies. Land-use included grasslands (GLD), grassland with trees (GLT, including some forests for grazing), and the integrated crop-livestock system (ICLS), and. (iv) The raw data were obtained from tables or extracted from the digitized graphs using WebPlotDigitizer (Version 3.8 for Desktop) in the publications.

The raw data were re-coded and re-arranged for meta-analysis based on the following principal rules:.

(i) Three levels of grazing intensities (heavy, moderate, and light grazing intensities) were selected based on most of the searched publications for this study. For some studies using numbers for grazing intensities, we re-coded the numbers in response to the three grazing levels. The more than three levels of the grazing intensities in some cases were re-coded as the three levels in terms of the grazed effects under the local conditions in these studies. For a few studies with more than three levels without un-grazing data, the lowest level (usually, the light or very light grazing) was regarded as the un-grazing level if the effects of the lowest grazing on soil properties were little based on the results in these studies.

(ii) For the paper with more than one site, if these sites were adjacent to each other or very close, only one data at one of the sites or averaged data across all sites to one value was selected after comparing the data from all sites to assure all data are relatively independent. However, if the distances among the sites were far (>46 km, an approximate distance of 25’ in latitude range) in one study, the data from different sites were regarded as relatively independent and included.

(iii) Data from one site but in different months during one year were averaged to one observation [1]. Because data from the same site in different years could be time-dependent (e.g., autocorrelation), the nearest year data were only included and these data from the other years at the same site were excluded.

(iv) The raw data for soil texture with percentage numbers were re-coded to the character type based on the Soil Texture Triangle by United States Department of Agriculture (USDA) Natural Resources Conservation Service (NRCS) (https://www.nrcs.usda.gov/wps/portal/nrcs/detail/soils). Then the sandy clay, sandy clay loam, sandy loam, loamy sandy, and sand were coded as “sandy” and other soil types were re-coded as “other”.

(v) The 0-10 cm depth usually reported for the top layer in selected studies and other increments were reported as 10-30 cm depth. Based on our data, we established sample increments of the 0-10 and 10-30 cm depths for BD, SOC, TN, C: N, pH, and P, and the 0-10 cm depth for other soil properties only (WC, NH_4_^+^, NO_3_^-^, K, PR, EC, CEC, MBC, and MBN) (S1 Table) due to lacking data. Data reported in different sub-depths within the above-defined increments were interpolated to the corresponding depth by summing the soil property data in sub-depths together.

(vi) Data for 33 variables (15 soil properties, 14 site information, and 4 publication information) in response to grazing intensities were summarized in S1 Dataset, and then were compiled as a dataset (S2 Dataset) for meta-analysis. Details of the variable names are presented in S1 Table. Other soil property data such as soil infiltration rate, soil hydraulic properties, soil aggregate properties, soil elements, exchangeable cations, soil microbial properties, and enzyme activities were excluded due to lacking enough quantity of publications (<30 lines of effect size).

Some variables were converted into those we needed by calculating using equations:

(i) For some cases with SOM but no SOC data, the SOM values were converted into SOC using the equation (their units should be same) [2]:

$SOC = SOM \times0.58$ (1)

(ii) In the studies with SOC stock (Mg ha^−1^) but no SOC (g kg^-1^) data, the SOM stock were converted into SOC using the equation [d = soil depth (m); BD = soil bulk density (Mg m^-3^)] [3]:

$SOC = SOC stock/(BD \times d)$ (2)

(iii) Within some studies with TN storage (Mg ha^−1^) without TN concentration (g kg^-1^) data, the TN storage was converted into TN concentration using the equation [d = soil depth (m); BD = soil bulk density (Mg m^-3^)] [4]:

$TN = TN storage/(BD \times d)$ (3)

For some MBC and MBN stocks, the same equation was used for converting these storages to concentration.

(iv) The C: N was calculated using the equation (units of SOC and TN must be the same):

$C:N = SOC/TN$ (4)

The equation was also used for calculating SOC or TN if the C: N and TN or SOC were given in some studies.

(v) The natural log of the response ratio was defined as “effect size” as a metric for the response of the soil property variables to grazing. For a given variable X, the effect size was calculated based on the equation:

$ESx=Ln \left( \frac{Xg}{Xug} \right)=LnXg-Ln(Xug)$ (5)

where *ES_x_* is the effect size of *X*; *X_g_* is the value of *X* in the grazed treatment group; *X_ug_* is the value of *X* in the control un-grazed group. One value of effect size was a log ratio of paired observations from the publication (i.e., an entry of paired observations). There was a total of 15 variables of soil property effect sizes (e.g., soil bulk density effect size, which was named as BD effect size) in this study. In total, the constructed dataset consisted of 2788 lines (or rows) of entries of paired observations for all 15 soil properties (S2 Dataset).

**Meta-Analysis Methods**

The mixed model method was used for meta-analysis using the MIXED procedure in SAS9.4 [5] in this study. Mixed models are appropriate for analyzing differences between groups of experiments when the groups are not expected to be internally homogeneous [6]. The mixed models can address the limitation of using randomization tests (resampling method) that cannot separate the two sources of variance (within-study sampling error and between-study variation in true effects) [6]. The 21 mixed models (15 for the 0-10 cm depth and 6 for the 10-30 cm depth) were built using the 21 soil property effect sizes as dependent variables, respectively. The grazing intensity (main effect), soil texture, land-use, grazing animal, latitude, altitude, precipitation, temperature, grazing period, and sampling year (the 9 variables were regarded as covariates for adjusting the mean effect sizes among the three grazing intensities) were taken as the fixed effects, and the study ID was used as the random effect in the 21 mixed models [7]. If the number of the effect size of one soil property under one grazing intensity < 10, the effect size was deleted and the data under the other two grazing intensities were used to build this mixed model (in this study, the numbers of effect sizes of 6 soil properties under the light grazing were less than 10, and the effect sizes were deleted). For the same reason, the data of land use (GLD, GLT, and ICLS) and animals (cattle, sheep, and mixed cattle and sheep) for some soil properties were not enough and deleted. The effect sizes were transformed when necessary for building the best-mixed models. The transformation method was determined using the Box-Cox method [8, 9] using the TRANSREG procedure in SAS9.4 [6]. Variance inflation factors (VIFs) were used to detect the presence of multicollinearity among the independent variables (A maximum VIF value above 10 is frequently taken as an indication that multicollinearity problem exists) [10] using the REG procedure in SAS9.4 [6]. The maximum VIF value in each model in this study was less than 10. The least square means (LS means) of effect sizes and their confidence intervals (CIs) of different grazing intensities from the mixed models were estimated using the variances of fixed and random effects based on the algorithm of SAS [11]. Based on this algorithm, the LS means were adjusted by covariates in the mixed models. The algorithm fit the effect size in the dataset S2 that included the unbalance data and incomplete data among the three grazing intensities due to limited publications. Because all transformed effect sizes were the monotonically increasing function of the original effect sizes, therefore, the LS means and CIs from the best models using the transformed effect sizes can be converted into the original values using the transformed equations. This could result in that the CIs were not symmetrical around their means. The estimated LS means and CIs of these effect sizes indicate the three grazing intensity effects on the soil properties. If the CI includes 0, the effect is not significant. If the CI excludes 0, the effect is significant (CI > 0 indicates the grazing has a significantly positive effect on the soil property, otherwise, the gazing significantly negatively impacts the soil property). The LS-means and CIs were reported as the percentage change estimated by (*e^ES^* − 1) × 100%. For other independent character variables, they impacted the effect sizes but we cannot use their LS means to explain their effects on soil properties because the dependent variables in the models were only the grazing effect sizes. However, we used the coefficients and p-values of the variables of fixed effects in the mixed models to interpret their impacts on soil property effect sizes, namely, effects of interaction between these variables and the grazing intensity on the soil properties. The positive (+) or negative (-) signs of coefficients of the independent variables as fixed effects in the mixed models indicate the positive or negative impacts of these variables on soil property effect sizes. These impacts are significant if the p-values of F-testing for coefficients of the independent variables < 0.10 (* represents the significant impacts in the tables in this study). However, the coefficients of independent variables cannot be used to interpret how many percentages they can increase or reduce the effect sizes of soil properties because most of the dependent variables (i.e., effect sizes of soil properties) were transformed using different algorithms based on the Box-Cox method when these best models were built.

Furthermore, the binomial logistic models were built to predict the probabilities of different grazing intensities resulting in overgrazing using SAS9.4 [6]. In these logistic models, the dependent variable was the grazing effect with binomial values “overgrazing” or “non-overgrazing”. The independent variables were grazing intensity, soil texture, land-use, grazing animal, effect size, latitude, altitude, precipitation, temperature, grazing period, and sampling year. Significance was determined at the α = 0.10 level for all statistical analysis because of different limited degrees of freedom for different soil property effect sizes (S1 Table) [12, 13].

**References**

1. Wang X, McConkey BG, VandenBygaart A, Fan J, Iwaasa A, Schellenberg M. Grazing improves C and N cycling in the Northern Great Plains: A meta-analysis. Sci Rep. 2016;6.

2. Mann L. Changes in soil carbon storage after cultivation. Soil Sci. 1986;142(5):279-88.

3. Lai L, Kumar S, Osborne S, Owens VN. Switchgrass impact on selected soil parameters, including soil organic carbon, within six years of establishment. Catena. 2018;163:288-96.

4. Chen Y, Li Y, Zhao X, Awada T, Shang W, Han J. Effects of grazing exclusion on soil properties and on ecosystem carbon and nitrogen storage in a sandy rangeland of Inner Mongolia, Northern China. Environ Manage. 2012;50(4):622-32.

5. SAS. SAS Institute. The SAS system for Windows. Release 9.4. SAS Inst., Cary, NC, USA. 2013.

6. Gurevitch J, Hedges LV. Statistical issues in ecological meta-analyses. Ecology. 1999;80(4):1142-9.

7. Sheu CF, Suzuki S. Meta-analysis using linear mixed models. Behav Res Methods Instrum Comput. 2001;33(2):102-7.

8. Box GE, Cox DR. An analysis of transformations. J Roy Stat Soc Ser B (Stat Method). 1964;26(2):211-52.

9. Box G, Cox D. An analysis of transformations revisited, rebutted. No. MRC-TSR-2288. Mathematics Research Center, Wisconsin University – Madison. 1981.

10. Kutner MH, Nachtsheim C, Neter J. Applied linear regression models. New York, NY, USA: McGraw-Hill Education; 2004.

11. SAS Institute Inc. SAS/STAT^®^9.2 User’s Guide, Second Edition. Cary, NC: SAS Institute Inc. 2009.

12. Royer I, Angers DA, Chantigny MH, Simard RR, Cluis D. Dissolved organic carbon in runoff and tile-drain water under corn and forage fertilized with hog manure. J Environ Qual. 2007;36(3):855-63.

13. Robert S, Torrie J, Dickey D. Principles and procedures of statistics: a biometrical approach. New York, NY, USA: McGraw-Hill; 1997.`
